# When Null Hypothesis Significance Testing Is Unsuitable for Research: A Reassessment

**DOI:** 10.3389/fnhum.2017.00390

**Published:** 2017-08-03

**Authors:** Denes Szucs, John P. A. Ioannidis

**Affiliations:** ^1^Department of Psychology, University of Cambridge Cambridge, United Kingdom; ^2^Meta-Research Innovation Center at Stanford and Department of Medicine, Department of Health Research and Policy, and Department of Statistics, Stanford University Stanford, CA, United States

**Keywords:** replication crisis, false positive findings, research methodology, null hypothesis significance testing, Bayesian methods

## Abstract

Null hypothesis significance testing (NHST) has several shortcomings that are likely contributing factors behind the widely debated replication crisis of (cognitive) neuroscience, psychology, and biomedical science in general. We review these shortcomings and suggest that, after sustained negative experience, NHST should no longer be the default, dominant statistical practice of all biomedical and psychological research. If theoretical predictions are weak we should not rely on all or nothing hypothesis tests. Different inferential methods may be most suitable for different types of research questions. Whenever researchers use NHST they should justify its use, and publish pre-study power calculations and effect sizes, including negative findings. Hypothesis-testing studies should be pre-registered and optimally raw data published. The current statistics lite educational approach for students that has sustained the widespread, spurious use of NHST should be phased out.

“What used to be called judgment is now called prejudice and what used to be called prejudice is now called a null hypothesis. In the social sciences, particularly, it is dangerous nonsense (dressed up as the “scientific method”) and will cause much trouble before it is widely appreciated as such.”

(Edwards, 1972; p.180.)

“…the mathematical rules of probability theory are not merely rules for calculating frequencies of random variables; they are also the unique consistent rules for conducting inference (i.e., plausible reasoning)”

(Jaynes, 2003; p. xxii).

## The replication crisis and null hypothesis significance testing (NHST)

There is increasing discontent that many areas of psychological science, cognitive neuroscience, and biomedical research (Ioannidis, 2005; Ioannidis et al., 2014) are in a crisis of producing too many false positive non-replicable results (Begley and Ellis, 2012; Aarts et al., 2015). This wastes research funding, erodes credibility and slows down scientific progress. Since more than half a century many methodologists have claimed repeatedly that this crisis may at least in part be related to problems with Null Hypothesis Significance Testing (NHST; Rozeboom, 1960; Bakan, 1966; Meehl, 1978; Gigerenzer, 1998; Nickerson, 2000). However, most scientists (and in particular psychologists, biomedical scientists, social scientists, cognitive scientists, and neuroscientists) are still near exclusively educated in NHST, they tend to misunderstand and abuse NHST and the method is near fully dominant in scientific papers (Chavalarias et al., 1990-2015). Here we provide an accessible critical reassessment of NHST and suggest that while it may have legitimate uses when there are precise quantitative predictions and/or as a heuristic, it should be abandoned as the *cornerstone* of research.

Our paper does not concern specifically the details of neuro-imaging methodology, many papers dealt with such details recently (Pernet and Poline, 2015; Nichols et al., 2016, 2017). Rather, we take a more general view in discussing fundamental problems that can affect any scientific field, including neuroscience and neuro-imaging. In relation to this it is important to see that non-invasive neuroscience data related to behavioral tasks cannot be interpreted if task manipulations did not work and/or behavior is unclear. This is because most measured brain activity changes can be interpreted in many different ways on their own (Poldrack, 2006; see Section 2.7 in Nichols et al., 2016). So, as most behavioral data are analyzed by NHST statistics NHST based inference from behavioral data also plays a crucial role in interpreting brain data.

## The origins of NHST as a weak heuristic and a decision rule

### NHST as a weak heuristic based on the *p*-value: Fisher

*p*-values were popularized by Fisher (1925). In the context of the current NHST approach Fisher *only* relied on the concepts of the null hypothesis (H_0_) and the *exact p-value* (hereafter p will refer to the *p*-value and “pr” to probability; see Appendix 1 in Supplementary Material for terms). He thought that experiments should aim to reject (or “nullify”; henceforth the name “null hypothesis”) H_0_ which assumes that the data demonstrates random variability according to some distribution around a certain value. Discrepancy from H_0_ is measured by a test statistic whose values can be paired with one or two-tailed *p*-values which tell us how likely it is that we would have found our data *or* more extreme data if H_0_ was really correct. Formally we will refer to the *p*-value as: pr(data or more extreme data|H_0_). It is important to realize that the *p*-value represents the “extremeness” of the data according to an imaginary data distribution assuming there is no bias in data sampling.

The late Fisher viewed the *exact p*-value as a *heuristic piece of inductive evidence* which gives an indication of the plausibility of H_0_ together with other available evidence, like effect sizes (see Hubbard and Bayarri, 2003; Gigerenzer et al., 2004). Fisher recommended that H_0_ can usually be rejected if *p* ≤ 0.05 but in his system there is no mathematical justification for selecting a particular *p*-value for the rejection of H_0_. Rather, this is up to the substantively informed judgment of the experimenter. Fisher thought that a hypothesis is demonstrable only when properly designed experiments “*rarely fail”* to give us statistically significant results (Gigerenzer et al., 1989, p. 96; Goodman, 2008). Hence, a single significant result should not represent a “scientific fact” but should merely draw attention to a phenomenon which seems worthy of further investigation including replication (Goodman, 2008). In contrast to the above, until recently replication studies have been very rare in many scientific fields; lack of replication efforts has been a particular problem in the psychological sciences (Makel et al., 2012), but this may hopefully change now with the wide attention that replication has received (Aarts et al., 2015).

### Neyman and Pearson: a decision mechanism optimized for the long-run

**Figure 1:**
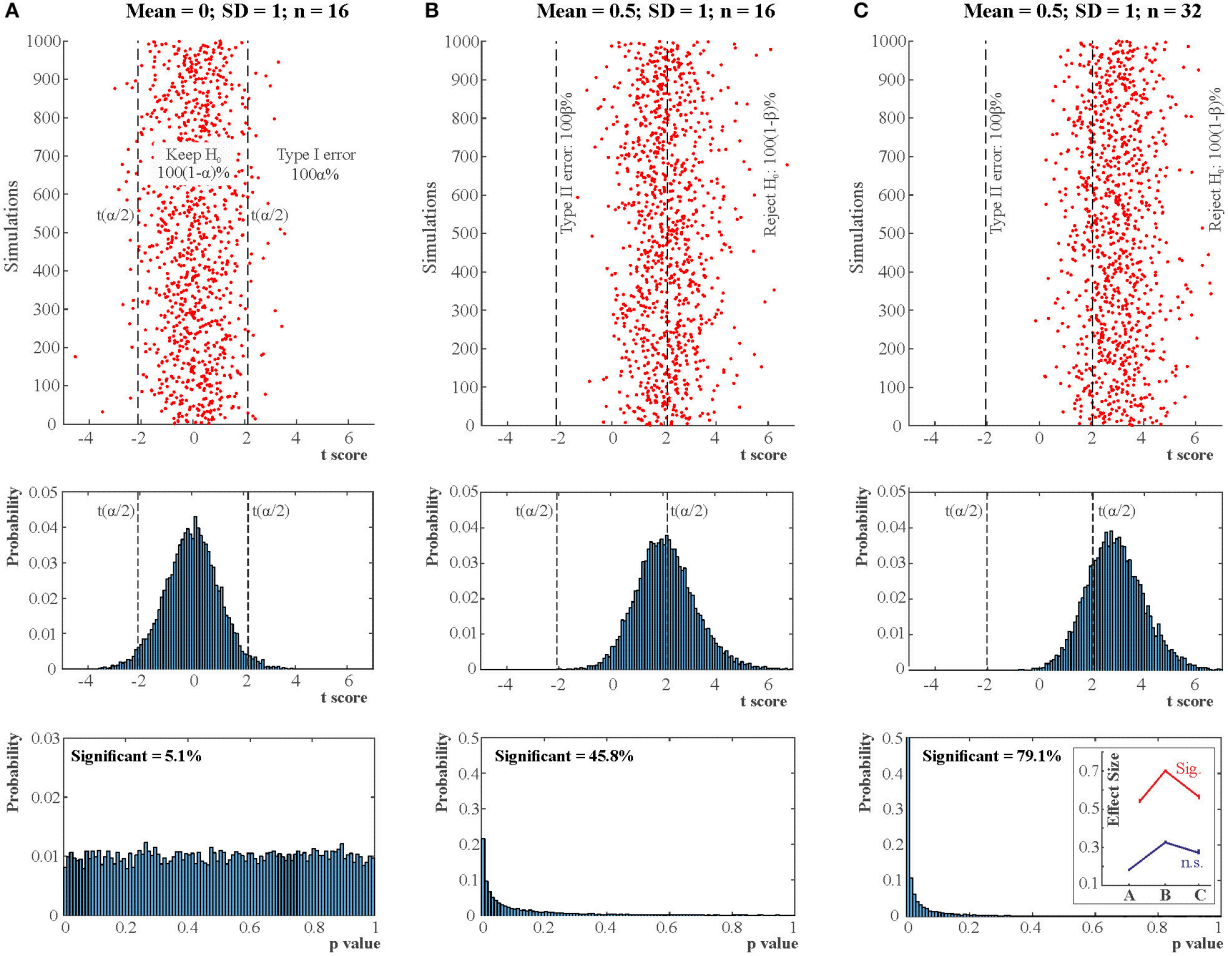
NHST concepts make sense in the context of a long run of studies. 3 × 10,000 studies with normally distributed data were simulated for 3 situations (**A:** True H_0_ situation: Mean = 0; *SD* = 1; *n* = 16. **B:** Mean = 0.5; *SD* = 1; *n* = 16; Power = 0.46. **C:** Mean = 0.5; *SD* = 1; n = 32; Power = 0.78.). One sample two-tailed *t-*tests determined whether the sample means were zero. The red dots in the top panels show t scores for 3 × 1,000 studies (not all studies are shown for better visibility). The vertical dashed lines mark the critical rejection thresholds for H_0_, t(α/2) for the two-tailed test. The studies producing a t statistic more extreme than these thresholds are declared statistically significant. The middle panels show the distribution of t scores for all 3 × 10,000 studies (bins = 0.1). The bottom panels show the distribution of *p*-values for all 3 × 10,000 studies (bins = 0.01) and state the proportion of significant studies. The inset in the bottom right panel shows the mean absolute effect sizes in standard deviation units for situations A-C from all significant (Sig.) and non-significant (n.s.) studies with 95% bias corrected and accelerated bootstrap confidence intervals (10,000 permutations). The real effect size was 0 in situation **(A)** and 0.5 in situations **(B,C)**. Note that the less is the power the more statistically significant studies overstate the effect size. Also note that *p*-values are randomly distributed and the larger is power the more right skewed is the distribution of *p*-values. In the true H_0_ situation the distribution of *p*-values is uniform between 0 and 1. See further explanation of this figure in Appendix 2 in Supplementary Material.

The concepts of the alternative hypothesis (H_1_), α, power, β, Type I, and Type II errors were introduced by Neyman and Pearson (Neyman and Pearson, 1933; Neyman, 1950) who set up a formal decision procedure motivated by industrial quality control problems (Gigerenzer et al., 1989). Their approach aimed to minimize the false negative (Type II) error rate to an acceptable level (β) and consequently to maximize power (1-β) *subject* to a bound (α) on false positive (Type I) errors (Hubbard and Bayarri, 2003). α can be set by the experimenter to an arbitrary value and Type-II error can be controlled by setting the sample size so that the required effect size can be detected (see Figure 1 for illustration). In contrast to Fisher, this framework does not use the *p*-value as a measure of evidence. We merely determine the critical value of the test statistic associated with α and reject H_0_ whenever the test statistic is larger than the critical value. The exact *p*-value is irrelevant because the sole objective of the decision framework is long-run error minimization and only the critical threshold but not the exact *p*-value plays any role in achieving this goal (Hubbard and Bayarri, 2003). Neyman and Pearson rejected the idea of inductive reasoning and offered *a reasoning*-*free inductive behavioral rule* to choose between two behaviors, accepting or rejecting H_0_, irrespective of the researcher's belief about whether H_0_ and H_1_ are true or not (Neyman and Pearson, 1933).

Crucially, the Neyman–Pearson approach is designed to work efficiently (Neyman and Pearson, 1933) in the context of long-run repeated testing (exact replication). Hence, there is a major difference between the *p*-value which is computed for a *single* data set and α, β, power, Type I, and Type II error which are so called “*frequentist”* concepts and they make sense in the context of *a long-run of many repeated experiments*. If we only run a single experiment all we can claim is that if we *had* run a long series of experiments we *would have had* 100α% false positives (Type I error) had H_0_ been true and 100β% false negatives (Type II error) had H_1_ been true *provided* we got the power calculations right. Note the conditionals.

In the Neyman–Pearson framework optimally setting α and β assures long-term decision-making efficiency in light of our costs and benefits by committing Type I and Type II errors. However, optimizing α and β is much easier in industrial quality control than in research where often there is no reason to expect a specific effect size associated with H_1_ (Gigerenzer et al., 1989). For example, if a factory has to produce screw heads with a diameter of 1 ± 0.01 cm than we know that we have to be able to detect a deviation of 0.01 cm to produce acceptable quality output. In this setting we know exactly the smallest effect size we are interested in (0.01 cm) and we can also control the sample size very efficiently because we can easily take a sample of a large number of screws from a factory producing them by the million assuring ample power. On the one hand, failing to detect too large or too small screws (Type II error) will result in our customers canceling their orders (or, in other industrial settings companies may deliver faulty cars or exploding laptops to customers exposing themselves to substantial litigation and compensation costs). On the other hand, throwing away false positives (Type I error), i.e., completely good batches of screws which we think are too small or too large, will also cost us a certain amount of money. Hence, we have a very clear scale (monetary value) to weigh the costs and benefits of both types of errors and we can settle on some rationally justified values of α and β so as to minimize our expenses and maximize our profit.

In contrast to such industrial settings, controlling the sample size and effect size and setting rational α and β levels is not that straightforward in most research settings where the true effect sizes being pursued are largely unknown and deciding about the requested size of a good enough effect can be very subjective. For example, what is the smallest difference of interest between two participant groups in a measure of “fMRI activity”? Or, what is the smallest difference of interest between two groups of participants when we measure their IQ or reaction time? And, even if we have some expectations about the “true effect size,” can we test enough participants to ensure a small enough β? Further, what is the cost of falsely claiming that a vaccine causes autism thereby generating press coverage that grossly misleads the public (Deer, 2011; Godlee, 2011)? What is the cost of running too many underpowered studies thereby wasting perhaps most research funding, boosting the number of false positive papers and complicating interpretation (Schmidt, 1992; Ioannidis, 2005; Button et al., 2013)? More often than not researchers do not know the “true” size of an effect they are interested in, so they cannot assure adequate sample size and it is also hard to estimate general costs and benefits of having particular α and β values. While some “rules of thumb” exist about what are small, modest, and large effects (e.g., Cohen, 1962, 1988; Jaeschke et al., 1989; Sedlmeier and Gigerenzer, 1989), some large effects may not be actionable (e.g., a change in some biomarker that is a poor surrogate and thus bears little relationship to major, clinical outcomes), while some small effects may be important and may change our decision (e.g., most survival benefits with effective drugs are likely to be small, but still actionable).

Given the above ambiguity, researchers fall back to the default α = 0.05 level with usually undefined power. So, the unjustified α and β levels completely discredit the originally intended “*efficiency”* rationale of the creators of the Neyman–Pearson decision mechanism (Neyman and Pearson, 1933).

### *P*-values are random variables and they correspond to standardized effect size measures

**Figure 2:**
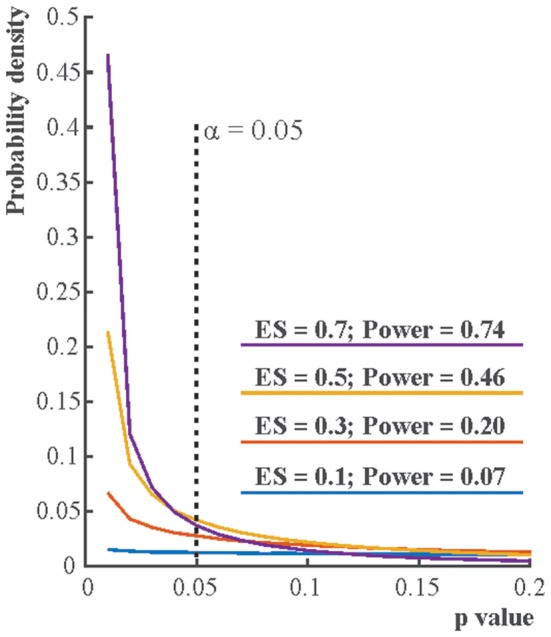
The distribution of *p*-values if the alternative hypothesis (H_1_) is true. Each line depicts the distribution of *p*-values resulting from one-sample two-tailed *t*-tests testing whether the sample mean was zero. Effect sizes (ES) indicate the true sample means for normally distributed data with standard deviation 1. For each effect size one million simulations were run with 16 cases in each simulation. The distribution of the *p*-value is becoming increasingly right skewed with increasing effect size and power. Note that α, the Type I error rate, is fix irrespective of what *p*-value is found in an experiment.

Contrary to the fact that in Figure 1 all 10,000 true H_0_ and 10,000 true H_1_ samples were simulated from identical H_0_ and H_1_ distributions, the t scores and the associated *p*-values reflect a dramatic spread. That is, *p*-values are best viewed as random variables which can take on a range of values depending on the actual data (Sterling, 1959; Murdoch et al., 2008). Consequently, it is impossible to tell from the outcome of a single (published) experiment delivering a statistically significant result whether a true effect exist. The only difference between the true H_0_ and true H_1_ situations is that when H_0_ is true in all experiments, the distribution of *p*-values is uniform between 0 and 1 whereas when H_1_ is true in all experiments *p*-values are more likely to fall on the left of the 0–1 interval, that is, their distribution becomes right skewed. The larger is the effect size and power the stronger is this right skew (Figure 2). This fact led to the suggestion that comparing this skew allows us to determine the robustness of findings in some fields by studying “p curves” (Hung et al., 1997; Simonsohn et al., 2014a,b). Hence, from this perspective, replication, and unbiased publication of all results (“positive” and “negative”) is again crucial if we rely on NHST because only then can they inform us about the distribution of *p*-values.

**Figure 3:**
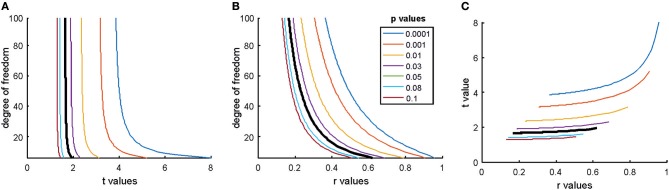
The relationship of the *p*-value, test statistic and effect sizes. **(A)** The relationship of t values, degrees of freedoms and *p*-values for Pearson correlation studies (df = n–2). **(B)** The relationship of Pearson correlation (r) values, degrees of freedoms, and *p*-values [r = t/sqrt(df + t^2^)]. **(C)** The relationship of *r*- and *t*-value pairs for each degree of freedom at various *p*-values. The bold black lines mark the usual significance level of α = 0.05. Note that typically only results which exceed the α = 0.05 threshold are reported in papers. Hence, papers mostly report exaggerated effect sizes.

Another point to notice is that both *p*-values and usual standardized effect size measures (Cohen's D, correlation values, etc.) are direct functions of NHST test statistics. Hence, for given degrees of freedom NHST test statistics, effect size measures and *p*-values will have non-linear correspondence as illustrated in Figure 3.

### NHST in its current form

The current NHST merged the approaches of Fisher and Neyman and Pearson and is often applied stereotypically as a “mindless null ritual” (Gigerenzer, 2004). Researchers set H_0_ nearly always “predicting” zero effect but do not quantitatively define H_1_. Hence, pre-experimental power cannot be calculated for most tests which is a crucial omission in the Neyman–Pearson framework. Researchers compute the *exact p*-value as Fisher did but also *mechanistically* reject H_0_ and accept the undefined H_1_ if *p* ≤ (α = 0.05) without flexibility following the *behavioral decision rule* of Neyman and Pearson. As soon as *p* ≤ α, findings have the supposed right to become a scientific fact defying the exact replication demands of Fisher and the belief neutral approach of Neyman and Pearson. Researchers also interpret the *exact p*-value and use it as a relative *measure of evidence* against H_0_, as Fisher did. A “*highly significant”* result with a small *p*-value is perceived as much stronger evidence than a weakly significant one. However, while Fisher was conscious of the weak nature of the evidence provided by the *p*-value (Wasserstein and Lazar, 2016), generations of scientists encouraged by incorrect editorial interpretations (Bakan, 1966) started to exclusively rely on the *p*-value in their decisions even if this meant neglecting their substantive knowledge: scientific conclusions *merged* with reading the *p*-value (Goodman, 1999).

## Neglecting the full context of NHST leads to confusions about the *p-*value

Most textbooks illustrate NHST by partial 2 × 2 tables (see Table 1) which fail to contextualize long-run conditional probabilities and fail to clearly distinguish between long-run probabilities and the *p*-value which is computed for a single data set (Pollard and Richardson, 1987). This leads to major confusions about the meaning of the *p*-value (see Appendix 2 in Supplementary Material).

**Table 1:** “pr” stands for probability.

		**True null effect (H_*0*_)**	**True positive effect (H_*1*_)**
*Pre-experiment probability of H_0_ and H_1_*	Long run of experiments	***pr(H**_0_**)***	***pr(H**_1_**)***
The conditional probability of having *this data or more extreme data* given that H_0_ is true	Single experiment	***p-*****value**	—
The conditional probability of having a significant test result given that H_0_ or H_1_ are true	Long run of experiments	**Alpha level (**α**) Type I error** False Positive False Alarm	**Power** = 1 −β True Positive Hit
The conditional probability of *not* having a significant test result given that H_0_ or H_1_ are true	Long run of experiments	1 – α = **Confidence level** True Negative Correct Rejection	β = 1 – Power **Type II error** False Negative Miss
*Post-experiment probability of H_*0*_ and H_*1*_ given a significant test result*	*Long run of experiments*	***FRP** pr(H_0_|significant result)*	***TRP** pr(H_1_|significant result)*

*NHST textbooks typically only present rows 3 and 4 of this table (Alpha level, Power, Confidence level and Type II error). We follow the NHST view and deal with long run probabilities only. Note that the p value does not fit this view as it does not have any long run interpretation besides that it is a random variable (Murdoch et al., 2008). The most important variables are bolded, familiar signal detection categories are also provided. NHST does not deal with the concepts in italics*.

First, both H_0_ and H_1_ have some usually unknown pre-study or “prior” probabilities, pr(H_0_) and pr(H_1_). Nevertheless, these probabilities may be approximated through extensive substantive knowledge. For example, we may know about a single published study claiming to demonstrate H_1_ by showing a difference between appropriate experimental conditions. However, in conferences we may have also heard about 9 highly powered but failed replication attempts very similar to the original study. In this case we may assume that the odds of H_0_:H_1_ are 9:1, that is, pr(H_1_) is 1/10. Of course, these pre-study odds are usually hard to judge unless we demand to see our colleagues' “null results” hidden in their drawers because of the practice of not publishing negative findings. Current scientific practices appreciate the single published “positive” study more than the 9 unpublished negative ones perhaps because NHST logic only allows for rejecting H_0_ but does not allow for accepting it *and* because researchers *erroneously* often think that the single published positive study has a very small, acceptable error rate of providing false positive statistically significant results which equals α, or the *p*-value. So, they often spuriously assume that the negative studies somehow lacked the sensitivity to show an effect while the single positive study is perceived as a well-executed sensitive experiment delivering a “conclusive” verdict rather than being a “lucky” false positive (Bakan, 1966). (See a note on pilot studies in Serious Underestimation of the Proportion of False Positive Findings in NHST).

NHST completely neglects the above mentioned pre-study information and exclusively deals with rows 2–4 of Table 1. NHST computes the one or two-tailed *p*-value for a particular data set assuming that H_0_ is true. Additionally, NHST logic takes long-run error probabilities (α and β) into account conditional on H_0_ and H_1_. These long-run probabilities are represented in typical 2 × 2 NHST contingency tables but note that β is usually unknown in real studies.

As we have seen, NHST *never* computes the probability of H_0_ and H_1_ being true or false, all we have is a decision mechanism hoping for the best individual decision in view of long-run Type I and Type II error expectations. Nevertheless, following the repeated testing logic of the NHST framework, for many experiments we can denote the *long-run probability* of H_0_ being true given a statistically significant result as False Report Probability (FRP), and the *long-run probability* of H_1_ being true given a statistically significant result as True Report Probability (TRP). FRP and TRP are represented in row 5 of Table 1 and it is important to see that they refer to completely *different conditional probabilities than* the *p*-value.

Simply put, the *p*-value is pretty much the only thing that NHST computes but scientists usually would like to know the probability of their theory being true or false in light of their data (Pollard and Richardson, 1987; Goodman, 1993; Jaynes, 2003; Wagenmakers, 2007). That is, researchers are interested in the post-experimental probability of H_0_ and H_1_. Most probably, for the reason that researchers do not get what they really want to see and the only parameter NHST computes is the *p*-value it is well-documented (Oakes, 1986; Gliner et al., 2002; Castro Sotos et al., 2007, 2009; Wilkerson and Olson, 2010; Hoekstra et al., 2014) that many, if not most researchers confuse FRP with the *p*-value or α and they also confuse the complement of *p*-value (1-p) or α (1-α) with TRP (Pollard and Richardson, 1987; Cohen, 1994). These confusions are of major portend because the difference between these completely different parameters is not minor, they can differ by orders of magnitude, the long-run FRP being much larger than the *p*-value under realistic conditions (Sellke et al., 2001; Ioannidis, 2005). The complete misunderstanding of the probability of producing false positive findings is most probably a key factor behind vastly inflated confidence in research findings and we suggest that this inflated confidence is an important contributor to the current replication crisis in biomedical science and psychology.

### Serious underestimation of the proportion of false positive findings in NHST

**Figure 4:**
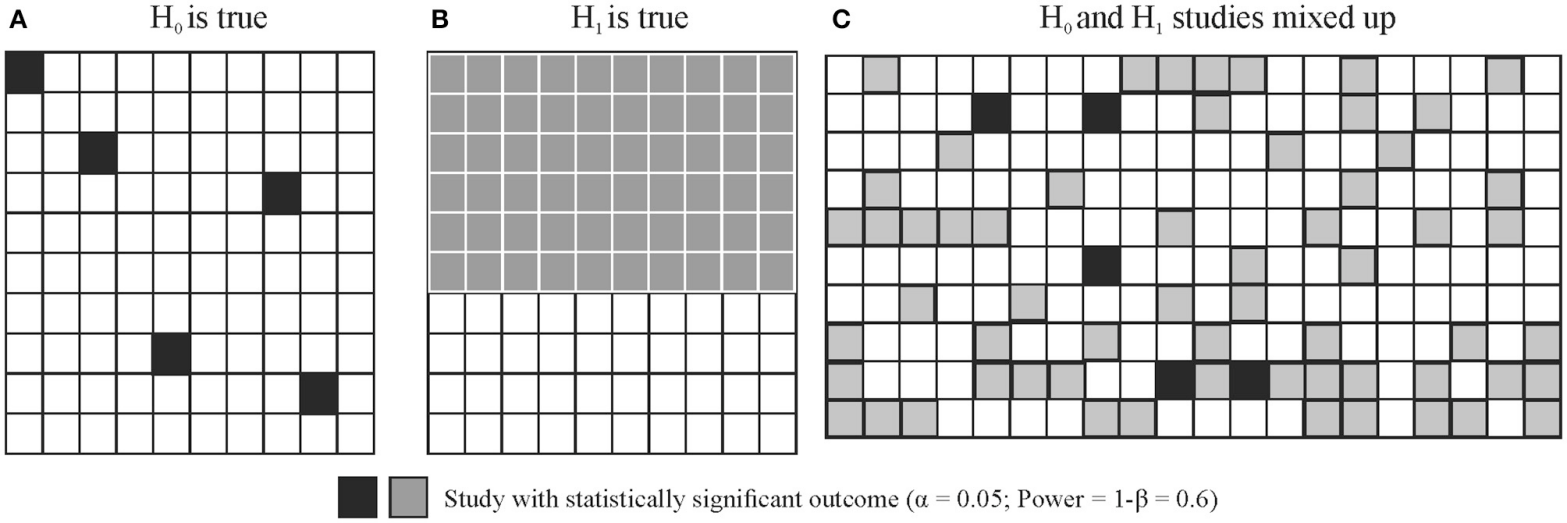
Illustration of long run False Positive Probability (FRP) and True Positive Probability (TRP) of studies. Let's assume that we run 2 × 100 studies, H_0_ is true in 100 studies and H_1_ is true in 100 studies with α = 0.05 and Power = 1−β = 0.6. **(A)** Shows the outcome of true H_0_ studies, 5 of the 100 studies coming up statistically significant. **(B)** Shows the outcome of true H_1_ studies, 60 of the 100 studies coming up statistically significant [note that realistically the 60 studies would be scattered around just as in panel **(A)** but for better visibility they are represented in a block]. **(C)** Illustrates that true H_0_ and true H_1_ studies would be indistinguishable. That is, researchers do not know which study tested a true H_0_ or true H_1_ situation (i.e., they could not distinguish studies represented by black and gray squares). All they know is whether the outcome of a particular study out of the 200 studies run was statistically significant or not. FRP is the ratio of false positive (H_0_ is true) statistically significant studies to all statistically significant studies: 5/65 = 0.0769. TRP is the ratio of truly positive (H_1_ is true) statistically significant studies to all statistically significant studies: 60/65 = 0.9231 = 1 − FRP = 1 − 0.0769.

Ioannidis (2005) has shown that most published research findings relying on NHST are likely to be false. The modeling supporting this claim refers to the long-run FRP and TRP which we can compute by applying Bayes' theorem (see Figure 4 for illustration, see computational details and further illustration in Appendix 3 in Supplementary Material). The calculations must consider α, the power (1-β) of the statistical test used, the pre-study probabilities of H_0_ and H_1_, and it is also insightful to consider bias (Berger, 1985; Berger and Delampady, 1987; Berger and Sellke, 1987; Pollard and Richardson, 1987; Lindley, 1993; Sellke et al., 2001; Sterne and Smith, 2001; Ioannidis, 2005).

While NHST neglects the pre-study odds of H_0_ and H_1_, these are crucial to take into account when calculating FRP and TRP. For example, let's assume that we run 200 experiments and in 100 studies our experimental ideas are wrong (that is, we test true H_0_ situations) while in 100 studies our ideas are correct (that is, we test true H_1_ situations). Let's also assume that the power (1-β) of our statistical test is 0.6 and α = 0.05. In this case in 100 studies (true H_0_) we will have 5% of results significant by chance alone and in the other 100 studies (true H_1_) 60% of studies will come up significant. FRP is the ratio of false positive studies to all studies which come up significant:

$$\begin{array}{l} FRP\;=\;\frac{False\;positives}{All\;statistically\;significant\;results}\\ =\;\frac{5\%\;of\;100\;studies}{5\%\;of\;100\;studies+60\%\;of\;100\;studies}\\ =\;\frac{5}{5\;+\;60}=\;\frac{5}{65}=0.0769 \end{array}$$

That is, we will have 5 false positives out of a total of 65 statistically significant outcomes which means that the proportion of false positive studies amongst all statistically significant results is 7.69%, higher than the usually assumed 5%. However, this example still assumes that we get every second hypothesis right. If we are not as lucky and only get every sixth hypothesis right then if we run 600 studies, 500 of them will have true H_0_ true situations and 100 of them will have true H_1_ situations. Hence, the computation will look like:

$$\begin{array}{l} FRP\;=\;\frac{False\;positives}{All\;statistically\;significant\;results}\\ =\;\frac{5\%\;of\;500\;studies}{5\%\;of\;500\;studies+60\%\;of\;100\;studies}\\ =\;\frac{25}{25\;+\;60}=\;\frac{25}{85}=0.2941 \end{array}$$

Hence, nearly 1/3 of all statistically significant findings will be false positives irrespective of the *p*-value. Note that this issue is basically the consequence of running multiple NHST tests throughout the whole literature and FRP can be considered the uncontrolled false discovery rate (FDR) across all studies run (see Section Family-Wise Error Rate (FWER) and FDR Correction in NHST).

Crucially, estimating pre-study odds is difficult, primarily due to the lack of publishing negative findings and to the lack of proper documentation of experimenter intentions before an experiment is run: We do not know what percent of the published statistically significant findings are lucky false positives explained *post-hoc* (Kerr, 1998) when in fact researchers could not detect the originally hypothesized effect and/or worked out analyses depending on the data (Gelman and Loken, 2014). However, it is reasonable to *assume* that only the most risk avoidant studies have lower H_0_:H_1_ odds than 1, relatively conservative studies have low to moderate H_0_:H_1_ odds (1–10) while H_0_:H_1_ odds can be much higher in explorative research (50–100 or even higher; Ioannidis, 2005).

The above H_0_:H_1_ assumptions are reasonable, as they are supported by empirical data in many different fields. For example, half or more of the drugs tested in large, late phase III trials show higher effectiveness against older comparators (H_0_:H_1_ = <1; Soares et al., 2005). Conversely, the vast majority of tested hypotheses in large-scale exploratory research reflect null effects, e.g., in the search of genetic variants associated with various diseases in the candidate gene era where investigators were asking hypotheses one or a few at a time (the same way that investigators continue to test hypotheses in most other biomedical and social science fields) yielded thousands of putative discovered associations, but only 1.2% of them were subsequently validated to be non-null when large-scale consortia with accurate measurements and rigorous analyses plans assessed them (Chanock et al., 2007; Ioannidis et al., 2011). Of the hundreds of thousands to many millions of variables assessed in current agnostic–omics testing, much less than 1% are likely to reflect non-null effects (H_0_:H_1_>>100). Lower rates of H_0_:H_1_ would be incompatible with logical considerations of how many variables are needed to explain all the variance of a disease or outcome risk.

Besides H_0_:H_1_ odds bias is another important determinant of FRP and TRP (Ioannidis, 2005). Whenever, H_0_ is not rejected findings have far more difficulty to be published and the researcher may feel that she wasted her efforts. Further, positive findings are more likely to get cited than negative findings (Kjaergard and Gluud, 2002; Jannot et al., 2013; Kivimäki et al., 2014). Consequently, researchers may often be highly biased to reject H_0_ and publish positive findings. Researcher bias affects FRP even if our NHST decision criteria, α and β, are formally unchanged. Ioannidis (2005) introduced the *u* bias parameter. The impact of u is that after some data tweaking and selective reporting (see Section NHST May Foster Selective Reporting and Subjectivity) u fraction of otherwise non-significant true H_0_ results will be reported as significant and u faction of otherwise non-significant true H_1_ results will be reported as significant. If u increases, FRP increases and TRP decreases. For example, if α = 0.05, power = 0.6, and H_0_:H_1_ odds = 1 then a 10% bias (u = 0.1) will raise FRP to 18.47%. A 20% bias will raise FRP to 26.09%. If H_0_:H_1_ odds = 6 then FRP will be 67.92%. Looking at these numbers the replication crisis does not seem surprising: using NHST very high FRP can be expected even with modestly high H_0_:H_1_ odds and moderate bias (Etz and Vandekerckhove, 2016). Hence, under realistic conditions FRP not only *extremely rarely* equals α or the *p*-value (and TRP extremely rarely equals 1-α and/or 1-*p*-value) but also, FRP is *much* larger than the generally assumed 5% and TRP is much lower than the generally assumed 95%. Overall, α or the *p*-value practically says nothing about the likelihood of our research findings being true or false.

At this point it is worth noting that it could be argued that unpublished pilot experiments may prompt us to run studies and hence, often H_0_:H_1_ odds would be lower than 1. However, unpublished pilot data often comes from small scale underpowered studies with high FRP, undocumented initial hypotheses and analysis paths. Hence, we doubt that statistically significant pilot results inevitably mean low H_0_:H_1_ odds.

### The neglect of power reinterpreted

In contrast to the importance of power in determining FRP and TRP, NHST studies tend to ignore power and β and emphasize α and low *p*-values. Often, finding a statistically significant effect erroneously seems to override the importance of power. However, statistical significance does not protect us from false positives. FRP can only be minimized by keeping H_0_:H_1_ odds and bias low and power high (Pollard and Richardson, 1987; Button et al., 2013; Bayarri et al., 2016). Hence, power is not only important so that we increase our chances to detect true effects but it is also crucial in keeping FRP low. While power in principle can be adjusted easily by increasing sample size, power in many/most fields of biomedical science and psychology has been notoriously low and the situation has not improved much during the past 50 years (Cohen, 1962; Sedlmeier and Gigerenzer, 1989; Rossi, 1990; Hallahan and Rosenthal, 1996; Button et al., 2013; Szucs and Ioannidis, 2017). Clearly, besides making sure that research funding is not wasted, minimizing FRP also provides very strong rationale for increasing the typically used sample sizes in studies.

## NHST logic is incomplete

### NHST misleads because it neglects pre-data probabilities

Besides often being subject to conceptual confusion and generating misleading inferences especially in the setting of weak power, NHST has further serious problems. NHST logic is based on the so-called *modus tollens* (denying the consequent) argumentation (see footnote in Appendix 4 in Supplementary Material): It sets up a H_0_ model and assumes that if the data fits this model than the test statistic associated with the data should not take more extreme values than a certain threshold (Meehl, 1967; Pollard and Richardson, 1987). If the test statistic contradicts this expectation then NHST assumes that H_0_ can be rejected and consequently its complement, H_1_ can be accepted. While this logic may be able to minimize Type I error in well-powered high-quality well-controlled tests (Section Neyman and Pearson: A decision Mechanism Optimized for the Long-Run), it is inadequate if we use it to decide about the truth of H_1_ in a single experiment, because there is always space for Type I and Type II error (Falk and Greenbaum, 1995). So, our conclusion is never certain and the only way to see how much error we have is to calculate the long-run FRP and TRP using appropriate α and power levels and prior H_0_:H_1_ odds. The outcome of the calculation can easily conflict with NHST decisions (see Appendix 4 in Supplementary Material).

### NHST neglects predictions under H_1_ facilitating sloppy research

NHST does not require us to specify exactly what data H_1_ would predict. Whereas, the Neyman–Pearson approach requires researchers to specify an effect size associated with H_1_ and compute power (1-β), in practice this is easy to *neglect* because the NHST machinery only computes the *p*-value conditioned on H_0_ and it is able to provide this result even if H_1_ is not specified at all. A widespread *misconception* flowing from the fuzzy attitude of NHST to H_1_ is that rejecting H_0_ allows for accepting a *specific* H_1_ (Nickerson, 2000). This is what most practicing researchers do in practice when they reject H_0_ and argue for their specific H_1_ in turn. However, NHST only computes probabilities conditional on H_0_ and it does not allow for the acceptance of either H_0_, a specific H_1_ or a generic H_1_. Rather, it only allows for the rejection of H_0_. Hence, if we reject H_0_ we will have no idea about how well our data fits a specific H_1_. This cavalier attitude to H_1_ can easily lead us astray even when contrasting H_0_ just with a single alternative hypothesis as illustrated by the invalid inference based on NHST logic in Table 2 (Pollard and Richardson, 1987).

**Table 2:** Potential NHST style argument (based on Pollard and Richardson, 1987).

**H_0_**	**Harold is American**
H_1_	Harold is not American
Model for H_0_	If Harold is American (H_0_), than he is *most probably not* a member of congress.
data	Harold *is* a member of congress.
pr(data or more extreme data|H_0_)	Very low
Inference	Because pr(data or more extreme data|H_0_) is very low, we reject H_0_ and accept H_1_ and conclude: Harold is *most probably not* American.

Our model says that if H_0_ is true, it is a *very rare* event that Harold is a member of congress. This rare event then happens which is equivalent to finding a small *p*-value. Hence, we conclude that H_0_ can be rejected and H_1_ is accepted (i.e., Harold *is* a member of congress and *therefore* he is not American.). However, if we carefully explicate all probabilities it is easy to see that we are being mislead by NHST logic. First, because we have absolutely no idea about Harold's nationality we can set pre-data probabilities of both H_1_ and H_0_ to 1/2, which means that H_0_:H_1_ odds are uninformative, 1:1. Then we can explicate the important conditional probabilities of the data (Harold *is* a member of congress) given the possible hypotheses. We can assign arbitrary but plausible probabilities:

$$\begin{array}{l} \text{pr}\left(\text{data}|{\text{H}}_{0}\right)=\text{pr}\left(\text{Harold is member of congress }|\text{ American}\right)\\ =10^{-7}\\ \text{pr}\left(\text{data}|{\text{H}}_{1}\right)=\text{pr}\left(\text{Harold is member of congress}|\text{not American}\right)\\ =0 \end{array}$$

That is, while the data is indeed rare under H_0_, its probability is actually zero under H_1_ (in other words, the data is very unlikely under both the null and the alternative models). So, even if *p* ≈ 0.0000001, it does not make sense to reject H_0_ and accept H_1_ because this data just cannot happen if H_1_ is true. If we only have these two hypotheses to choose from then it only makes sense to accept H_0_ because the data is still possible under H_0_ (Jaynes, 2003). In fact, using Bayes' theorem we can formally show that the probability of H_0_ is actually 1 (Appendix 5 in Supplementary Material).

In most real world problems multiple alternative hypotheses compete to explain the data. However, by using NHST we can only reject H_0_ and argue for *some* H_1_ without any formal justification of why we prefer a particular hypothesis whereas it can be argued that it only makes sense to reject any hypothesis if another one better fits the data (Jaynes, 2003). We only have qualitative arguments to accept a specific H_1_ and the exclusive focus on H_0_ makes unjustified inference too easy. For example, if we assume that H_0_ predicts normally distributed data with mean 0 and standard deviation 1 then we have endless options to pick H_1_ (Hubbard and Bayarri, 2003): Does H_1_ imply that the data have a mean other than zero, the standard deviation other than 1 and/or does it represent non-normally distributed data? NHST allows us to consider any of these options *implicitly* and then accept one of them *post-hoc* without any quantitative justification of why we chose that particular option. Further, merging all alternative hypotheses into a single H_1_ is not only too simplistic for most real world problems but it also poses an “inferential double standard” (Rozeboom, 1960): The procedure pits the well-defined H_0_ against a potentially infinite number of alternatives.

Vague H_1_ definitions (the lack of quantitative predictions) enable researchers to avoid the falsification of their favorite hypotheses by intricately redefining them (especially in fields such as psychology and cognitive neuroscience where theoretical constructs are often vaguely defined) and never providing any definitive assessment of the plausibility of a favorite hypothesis in light of credible alternatives (Meehl, 1967). This problem is reflected in papers aiming at the mere demonstration of often little motivated significant differences between conditions (Giere, 1972) and *post-hoc* explanations of likely unexpected but statistically significant findings. For example, neuroimaging studies often attempt to explain why an fMRI BOLD signal “deactivation” happened instead of a potentially more reasonable looking “activation” (or, vice versa). Most such findings may be the consequence of the data randomly deviating into the wrong direction relative to zero between-condition difference. Even multiple testing correction will not help such studies as they still rely on standard NHST just with adjusted α thresholds. Similarly, patient studies often try to explain an unexpected difference between patient and control groups (e.g., the patient group is “better” on a measure or shows “more” or “less” brain activation) by some kind of “compensatory mechanism.” In such cases what happens is that “*the burden of inference has been delegated to the statistical test*,” indeed, and simply because *p* ≤ α odd looking observations and claims are to be trusted as scientific facts (Bakan, 1966, p. 423; Lykken, 1968).

Finally, paradoxically, when real life practicing researchers achieve their “goal” and successfully reject H_0_ they may be left in complete existential vacuum because during the rejection of H_0_ NHST “*saws off its own limb”* (Jaynes, 2003; p. 524): If we manage to reject H_0_ then it follows that pr(data or more extreme data|H_0_) is useless because H_0_ is not true. Thus, we are left with nothing to characterize the probability of our data in the real world; we will not know pr(data|H_1_) for example, because H_1_ is formally undefined and NHST never tells us anything about it. In light of these problems Jaynes (2003) suggested that the NHST framework addresses an ill-posed problem and provides invalid responses to questions of statistical inference.

It is noteworthy that some may argue that Jaynes's argument is formally invalid as the NHST approach can be used to reject a low probability H_0_ *in theory*. However, recall that (1) NHST does not deliver final objective theoretical decisions, there is no theoretical justification for any α thresholds marking a boundary of informal surprise and NHST merely aims to minimize Type I error on the long run (and in fact, Neyman and Pearson (1933) considered their procedure a theory-free decision mechanism and Fisher considered it a heuristic). (2) NHST can only reject H_0_ (heuristically or in a theory-free manner) and (3) cannot provide support for any H_1_. We could also add that many practicing biomedical and social scientists may not have clear quantitative predictions under H_0_ besides expecting to reject a vague null effect (see Section NHST in Sciences with and without Exact Quantitative Predictions for the difference between sciences with and without exact predictions). Hence, their main (ultimate) objective of using NHST is often actually not the *falsification* of the *exact* theoretical predictions of a well-defined theory (H_0_). Rather, they are more interested in arguing in favor of an alternative theory. For example, with a bit of creativity fMRI “activation” in many different (perhaps *post-hoc* defined) ROIs can easily be “explained” by some theory when H_0_ (“no activation”) is rejected in any of the ROIs. However, supporting a specific alternative theory is just not possible in the NHST framework and in this context Jaynes' comment is perfectly valid: NHST provides an ill-defined framework, after rejecting H_0_ real-world researchers have no formal hypothesis test outcomes to support their “positive” arguments.

### NHST is unsuitable for large datasets

In consequence of the recent ‘big data’ revolution access to large databases has increased dramatically potentially increasing power tremendously (though, large data sets with many variables are still relatively rare in neuroscience research). However, NHST leads to worse inference with large databases than with smaller ones (Meehl, 1967; Khoury and Ioannidis, 2014). This is due to how NHST tests statistics are computed, the properties of real data and to the lack of specifying data predicted by H_1_ (Bruns and Ioannidis, 2016).

Most NHST studies rely on nil null hypothesis testing (Nickerson, 2000) which means that H_0_ expects a true mean difference of exactly zero between conditions with some variation around this true zero mean. Further, NHST machinery guarantees that we can detect any tiny irrelevant effect sizes if sample size is large enough. This is because test statistics are typically computed as the ratio of the relevant between condition differences and associated variability of the data weighted by some function of the sample size [difference/variability × f(sample size)]. The *p*-value is smaller if the test statistic is larger. Thus, the larger is the difference between conditions and/or the smaller is variability and/or the larger is the sample size the larger is the test statistic and the smaller is the *p*-value (see Figure 3 for examples). Consequently, by increasing sample size enough it is guaranteed that H_0_ can be rejected even with miniature effect sizes (Ziliak and McCloskey, 2008).

Parameters of many real data sets are much more likely to differ than to be the same for reasons completely unrelated to our hypotheses (Meehl, 1967, 1990; Edwards, 1972). First, many psychological, social and biomedical phenomena are extremely complex reflecting the contribution of very large numbers of interacting (latent) factors, let it be at the level of society, personality or heavily networked brain function or other biological networks (Lykken, 1968; Gelman, 2015). Hence, if we select any two variables related to these complex networks most probably there will be some kind of at least remote connection between them. This phenomenon is called “crud factor” Meehl (1990) or “ambient correlational noise” (Lykken, 1968) and it is unlikely to reflect a causal relationship. In fact some types of variables, such as intake of various nutrients and other environmental exposures are very frequently correlated among themselves and with various disease outcomes without this meaning that they have anything to do with causing disease outcomes (Patel and Ioannidis, 2014a,b). Second, unlike in physical sciences it is near impossible to control for the relationship of all irrelevant variables which are correlated with the variable(s) of interest (Rozeboom, 1960; Lykken, 1968). Consequently, there can easily be a small effect linking two randomly picked variables even if their statistical connection merely communicates that they are part of a vast complex interconnected network of variables. Only a few of these tiny effects are likely to be causal and of any portend (Siontis and Ioannidis, 2011).

The above issues have been demonstrated empirically and by simulations. For example, Bakan (1966; see also Berkson, 1938; Nunnally, 1960; Meehl, 1967) subdivided the data of 60,000 persons according to completely arbitrary criteria, like living east or west of the Mississippi river, living in the north or south of the USA, etc. and found all tests coming up statistically significant. Waller (2004) examined the personality questionnaire data of 81,000 individuals to see how many randomly chosen directional null hypotheses can be rejected. If sample size is large enough, 50% of directional hypothesis tests should be significant irrespective of the hypothesis. As expected, nearly half (46%) of Waller's (2004) results were significant. Simulations suggest that in the presence of even tiny residual confounding (e.g., some omitted variable bias) or other bias, large observational studies of null effects will generate results that may be mistaken as revealing thousands of true relationships (Bruns and Ioannidis, 2016). Experimental studies may also suffer the same problem, if they have even minimal biases.

### NHST in sciences with and without exact quantitative predictions

Due to the combination of the above properties of real-world data sets and statistical machinery theory testing radically differs in sciences with exact and non-exact quantitative predictions (Meehl, 1967). In physical sciences increased measurement precision and increased amounts of data increase the difficulties a theory must pass before it is accepted. This is because theoretical predictions are well-defined, numerically precise and it is also easier to control measurements (Lykken, 1968). Hence, NHST may be used to aim to falsify exact theoretical predictions. For example, a theory may predict that a quantity should be let's say 8 and the experimental setup can assure that really only very few factors influence measurements—these factors can then be taken into account during analysis. Hence, increased measurement precision will make it easier to demonstrate a departure from numerically exact predictions. So, a “five sigma” deviation rule may make good sense in physics where precise models are giving precise predictions about variables.

In sciences using NHST without clear numerical predictions the situation is the opposite of the above, because NHST does not demand the exact specification of H_1_, so theories typically only predict a fairly vague “*difference”* between groups or experimental conditions rather than an exact numerical discrepancy between measures of groups or conditions. However, as noted, groups are actually likely to differ and if sample size increases and variability in data decreases it will become easier and easier to reject any kind of H_0_ when following the NHST approach. In fact, with precise enough measurements, large enough sample size and repeated “falsification” attempts H_0_ is guaranteed to be rejected on the long run (see Section The Rejection of H_0_ is Guaranteed on the Long-Run) even if the underlying processes generating the data in two experimental conditions are exactly the same. Hence, ultimately any H_1_ can be accepted, claiming support for any kind of theory. For example, in an amusing demonstration Carver (1993) used Analysis of Variance to re-analyze the data of Michaelson and Morley (1887) who came up with a “dreaded” null finding and based on this they suggested that the speed of light was constant (H_0_) thereby providing empirical support for Einstein's theory of relativity. Carver (1993) found that that the speed of light was actually not constant at *p* < 0.001. The catch? The effect size as measured by Eta^2^ was 0.005. While some may feel that Einstein's theory has now been falsified, perhaps it is also worth considering that here the statistically significant result is essentially insignificant. This example also highlights the fact we are not arguing against the Popperian view of scientific progress by falsifying theories. Rather, we discuss why NHST is a very imperfect method for this falsification (see further arguments as well).

A typical defense of NHST may be that we actually may not want to increase power endlessly, just as much as we still think that it allows us to detect reasonable effect sizes (Giere, 1972). For example, equivalence testing may be used to reject the hypothesis that a meaningfully large effect exist (e.g., Wellek, 2010) or researchers may check for the sign of expected effects (Gelman and Tuerlinckx, 2000). However, because typically only statistically significant data is published, published studies most probably exaggerate effect sizes. So, estimating true (expected) effect sizes is very difficult. A more reasoned approach may be to consider explicitly what the consequences (“costs”) are of a false-positive, true-positive, false-negative, and true-negative result. Explicit modeling can suggest that the optimal combination of Type 1 error and power may need to be different depending on what these assumed costs are (Djulbegovic et al., 2014). Different fields may need to operate at different optimal ratios of false-positives to false-negatives (Ioannidis et al., 2011).

### NHST may foster selective reporting and subjectivity

Because NHST never evaluates H_1_ formally and it is fairly biased toward the rejection of H_0_, reporting bias against H_0_ can easily infiltrate the literature even if formal NHST parameters are fixed (see Section Serious Underestimation of the Proportion of False Positive Findings in NHST about the “u” bias parameter). Overall, a long series of exploratory tools and questionable research practices are utilized in search for statistical significance (Ioannidis and Trikalinos, 2007; John et al., 2012). Researchers can influence their data during undocumented analysis and pre-processing steps and by the mere choice of structuring the data (constituting *researcher degrees of freedom*; Simmons et al., 2011). This is particularly a problem in neuroimaging where the complexity and idiosyncrasy of analyses is such that it is usually impossible to replicate exactly what happened and why during data analysis (Kriegeskorte et al., 2009; Vul et al., 2009; Carp, 2012). Another term that has been used to describe the impact of diverse analytical choices is “vibration of effects” (Ioannidis, 2008). Different analytical options, e.g., choice of adjusting covariates in a regression model can result in a cloud of results, instead of a single result, and this may entice investigators to select a specific result that is formally significant, while most analytical options would give non-significant results or even results with effects in the opposite direction (“Janus effect”; Patel et al., 2015). Another common mechanism that may generate biased results with NHST is when investigators continue data collection and re-analyse the accumulated data sequentially without accounting for the penalty induced by this repeated testing (DeMets and Lan, 1994; Goodman, 1999; Szucs, 2016). The unplanned testing is usually undocumented and researchers may not even be conscious that it exposes them to Type I error accumulation. Bias may be the key explanation why in most biomedical and social science disciplines, the vast majority of published papers with empirical data report statistically significant results (Kavvoura et al., 2007; Fanelli, 2010; Chavalarias et al., 1990-2015). Overall, it is important to see that NHST can easily be infiltrated by several undocumented subjective decisions. (Bayesian methods are often blamed such subjectivity, see Section Teach Alternative Approaches Seriously.)

### The rejection of H_0_ is guaranteed on the long-run

If H_0_ is true, with α = 0.05, 5% of our tests will be statistically significant on the long-run. The riskier experiments we run, the larger are H_0_:H_1_ odds and bias and the larger is the long-run FRP. For example, in a large laboratory with 20 post-docs and PhD students, each person running 5 experiments a year implementing 10 significance tests in each experiment we can expect 20 × 5 × 10 × 0.05 = 50 [usually publishable] false results a year at α = 0.05 if H_0_ is true. Coupled with the fact that a large number of unplanned tests may be run in each study (Simmons et al., 2011; Gelman and Loken, 2014) and that negative results and failed replications are often not published, this leads to “*unchallenged fallacies”* clogging up the research literature (Ioannidis, 2012; p1; Sterling, 1959; Bakan, 1966; Sterling et al., 1995). Moreover, such published false positive true H_0_ studies will also inevitably overestimate the effect size of the non-existent effects or of existent, but unimportantly tiny, effects (Schmidt, 1992, 1996; Sterling et al., 1995; Ioannidis, 2008). These effects may even be confirmed by meta-analyses, because meta-analyses typically are not able to incorporate unpublished negative results (Sterling et al., 1995) and they cannot correct many of the biases that have infiltrated the primary studies. For example, such biases may result in substantial exaggeration of measured effect sizes in meta-analyses (see e.g., Szucs and Ioannidis, 2017).

Given that the predictions of H_1_ are rarely precise and that theoretical constructs in many scientific fields (including psychology and cognitive neuroscience) are often poorly defined (Pashler and Harris, 2012), it is easy to claim support for a popular theory with many kinds of data falsifying H_0_ even if the constructs measured in many papers are just very weakly linked to the original paper, or not linked at all. Overall, the literature may soon give the impression of a steady stream of replications throughout many years. Even when “negative” results appear, citation bias may still continue to distort the literature and the prevailing theory may continue to be based on the “positive” results. Hence, citation bias may maintain prevailing theories even when they are clearly false and unfounded (Greenberg, 2009).

### NHST does not facilitate systematic knowledge integration

Due to high FRP the contemporary research literature provides statistically significant “evidence” for nearly everything (Schoenfeld and Ioannidis, 2012). Because NHST emphasizes all or none *p*-value based decisions rather than the magnitude of effects, often only *p*-values are reported for critical tests, effect size reports are often missing and interval estimates and confidence intervals are not reported. In an assessment of the entire biomedical literature in 1990–2015, 96% of the papers that used abstracts reported at least some *p*-value below 0.05, while only 4% of a random sample of papers presented consistently effect sizes with confidence intervals (Chavalarias et al., 1990-2015). However, oddly enough, the main NHST “measure of evidence,” the *p*-value cannot be compared across studies. It is a frequent *misconception* that a lower *p*-value always means stronger evidence irrespective of the sample size and effect size (Oakes, 1986; Schmidt, 1996; Nickerson, 2000). Besides the non-comparable *p*-values, NHST does not offer any *formal* mechanism for systematic knowledge accumulation and integration (Schmidt, 1996) unlike Bayesian methods which can take such pre-study information into account. Hence, we end up with many fragmented studies which are most often unable to say anything formal about their favorite H_1_s (accepted in a qualitative manner). Methods do exist for the meta-analysis of *p*-values (see e.g., Cooper et al., 2009) and these are still used in some fields. However, practically such meta-analyses still say nothing about the magnitude of the effect size of the phenomenon being addressed. These methods are potentially acceptable when the question is whether there is any non-null signal among multiple studies that have been performed, e.g., in some types of genetic associations where it is taken for granted that the effect sizes are likely to be small anyhow (Evangelou and Ioannidis, 2013).

### Family-wise error rate (FWER) and FDR correction in NHST

An increasingly important problem is that with the advent of large data sets researchers can use NHST to test multiple, related hypotheses. For example, this problem routinely appears in neuro-imaging where a large amount of non-independent data points are collected and then the same hypothesis test may be run on tens of thousands of observations, for example, from a brain volume, or from 256 electrodes placed on the scalp, each electrode recording voltage 500 times a second. Analysis procedures that generate different views of data (e.g., time-frequency or independent component analyses) may further boost the amount of tests to be run.

Regarding these multiple testing situations, a group of statistical tests which are somehow related to each other can be defined as a “family of comparisons.” The probability that a family of comparisons contains at least one false positive error is called the family wise error rate (FWER). If the repeated tests concern independent data sets where H_0_ is true than the probability of having at least one Type I error in k independent tests, each with significance level α, is α_TOTAL_ = 1 - (1 - α)^k^. For example if k = 1, 2, 3, 4, 5, and 10 than α_TOTAL_ is 5, 9.75, 14.26, 18.55, 22.62, and 40.13%, respectively (see Curran-Everett, 2000; Szucs, 2016 for graphical illustrations and simulations for non-independent data).

There are numerous procedures which can take multiple testing into account by correcting *p*-values. The simplest of these procedures is Bonferroni correction which computes an adjusted *p*-value threshold as α/n where α is the statistical significance threshold for a single test and n is the number of tests run. Hence, if we run 5 tests which can be defined as a family of tests and our original α is 0.05 then the Bonferroni corrected adjusted α level is 0.05/5 = 0.01. Any *p*-values above this threshold should not be considered to demonstrate statistically significant effects. Besides the Bonferroni correction there are other alternative methods of FWER correction, like the Tukey Honestly Significant Difference test, the Scheffe test, Holm's method, Sidak's method; Hochberg's method, etc. Some of these corrections also take the dependency (non-independence) of tests into account (see e.g., Shaffer, 1995; Nichols and Hayasaka, 2003 for review).

FWER control is a conservative procedure in keeping Type I error rate low but it also sacrifices power increasing Type II error. An alternative to FWER control is False Discovery Rate (FDR) control which allows more Type I errors but assures higher power. Using the same logic as the computation of FRP discussed before, FDR control considers the estimated proportion of false positive statistically significant findings amongst all statistically significant findings (i.e., the proportion of erroneously rejected null hypotheses out of all rejected null hypotheses; Benjamini and Hochberg, 1995). FDR computation is illustrated by Table 3. If we run M hypothesis tests then a certain number of them are likely to test true null effects (M_0_ in Table 3) and some other number of them are likely to test non-null effects with true alternative hypotheses (M_1_ in Table 3). Depending on our α level and power (1-β), a certain number of the M_0_ and M_1_ tests will reject the null hypothesis (FP and TN, respectively, see Table 3 for abbreviations) while some other number of them will not reject the null hypothesis (TN and FN, respectively). If we know the exact numbers in Table 3 then the proportion of false positive statistically significant findings can be computed as the ratio of false positive results to all statistically significant results: Q = FP/(FP + TP) = FP/R (assuming that R≠ 0).

**Table 3:** Illustrating the logic behind FDR computation.

	**Null hypothesis is true**	**Alternative hypothesis is true**	**Sums**
H_0_ is rejected (statistically significant outcome)	FP = False Positives 45 (if α = 0.05; 900·0.05 = 45)	TP = True Positives 60 (if power = 0.6; 100·0.6 = 60)	R 105
H_0_ is not rejected (statistically non-significant outcome)	TN = True Negatives 855 (if α = 0.05; 900·0.95 = 855)	FN = False Negatives 40 (if β = 0.4; 100·0.4 = 40)	M – R 895
Sums	M_0_ 900	M_1_ 100	M 1000

*H_0_ (leftmost column) stands for the null hypothesis. The proportion of false positive statistically significant test outcomes to all statistically significant test outcomes is Q = FP/(FP + TP) = FP/R (R≠0). The numbers give an example for the case when α = 0.05 (so, FWER = α = 0.05) and β = 0.4, so Power = 1 − β = 0.6. In the example we run 1,000 null hypothesis tests. We test 9 times as many true null situations than situations with true alternative hypotheses (that is, every 10th of our experimental ideas are correct). In this case Q = 45/105 = 0.4286. That is, 42.86% of statistically significant results will be false positives. FDR = Q · pr(R > 0) = Q · [α · (M_0_/M) + Power · (M_1_/M)] = 0.045. If we only test true null effects then Q = false positives/all significant results = α·M / α·M = 1; and FDR = Q · pr(R>0) = Q · α = α = FWER [pr(R>0) = α because all significant results are coming from true null situations]. Note that in real research only R, M, and M-R are known whereas M_0_, M_1_, and Q are not known*.

Of course, in real research settings we do not know how many of our tests test true null effects and we only know how many tests we run and how many of them return statistically significant and non-significant results. So Q can be considered a random variable. However, as Q cannot be controlled directly FDR is defined as the expected value of the proportion of false positive errors: FDR = E[FP/R|R > 0] · pr(R > 0), a variable which can be controlled (see Benjamini and Hochberg, 1995; Curran-Everett, 2000; Nichols and Hayasaka, 2003; Bennett et al., 2009; Benjamini, 2010; Goeman and Solari, 2014). Some FDR estimation procedures can also factor in dependency between tests (Benjamini and Yekutieli, 2001).

In contrast to FDR, using the notation in Table 3, FWER can be expressed as FWER = pr(FP≥1) = 1 − pr(FP = 0) that is, the probability that there is at least one false positive Type I error in a family of observations. If the null hypothesis is true in all tests we run then FDR = FWER while if there are situations with true alternative hypotheses then FDR < FWER (see Table 3 for example). Also, various other FDR and FWER measures can be derived (see the above cited reviews). It can be argued that controlling FDR is more useful in research where a very large number of tests are carried out routinely, like neuro-imaging or genetics but less useful in behavioral psychological and social science research where fewer hypotheses may be tested at any one time and accepting any single hypothesis as statistically significant may have large impact on inferences (Gelman et al., 2012). This last statement is also true for behavioral data used to support the interpretation of neuro-imaging findings.

Most relevant to our paper, both FWER and FDR error rate corrections are based on the same NHST procedure. That is, they do not modify the procedure in any ways other than aiming to decrease Type I error toward initially expected levels when multiple NHST tests are run. That is, these methods can help in constraining the number of random findings when a single hypothesis is tested simultaneously in many data points (e.g., voxels) but do nothing to protect against many of the other problems discussed in this paper (e.g., generating a high amount of false positives across the literature; being sensitive to undocumented biasing procedures; neglecting predictions under H_1_; not providing probability statements for H_1_; neglecting pre-data probabilities; being unable to effectively integrate study results). These problems are valid even when just one single NHST test is run. In addition, empirical analyses of large fMRI data sets found that the most popular fMRI analysis software packages implemented erroneous multiple testing corrections and hence, generate much higher levels of false positive results than expected (Eklund et al., 2012, 2016). This casts doubts on a substantial part of the published fMRI literature. Further, Carp (2012) reported that about 40% of 241 relatively recent fMRI papers actually did not report having used multiple testing correction. So, a very high percentage of fMRI literature may have been exposed to high false positive rates either multiple correction was used or not (see also (Szucs and Ioannidis, 2017) on statistical power).

In the NHST framework the multiple comparison problem is exacerbated by the fact that we may test a very large number of precise null hypotheses (Neath and Cavanaugh, 2006), often without much theoretical justification (e.g., in many explorative whole brain analyses). However, as the H_0_:H_1_ odds may be high the NHST mechanism may produce a very large number of falsely significant results. Similarly high numbers of false alarms are produced under realistic conditions even if some rudimentary model is used for the data (e.g., expecting positive or negative difference between conditions; Gelman and Tuerlinckx, 2000). In contrast, while currently there is no standard way to correct for multiple comparisons with Bayesian methods, Bayesian methods have been shown to be more conservative than NHST in some situations (Gelman and Tuerlinckx, 2000) and they offer various methods for correcting for multiple comparisons (e.g., Westfall et al., 1997; Gelman et al., 2014). In addition, Bayesian methods are strongly intertwined with explicit model specifications. These models can then be used to generate simulated data and to study model response behavior. This may offer a way to judge the reasonableness of analyses offering richer information than NHST accept/reject decisions (Gelman et al., 2014). The challenge of course is the development of models. However, below we argue that efficient model development can only happen if we refocus our efforts on understanding data patters from the testing of often very vaguely defined hypotheses. In addition, Bayesian methods are also able to formally aggregate data from many experiments (e.g., adding data serially and by hierarchical models; Gelman et al., 2014). This can further maximize large-scale joint efforts for better model specifications.

## The state of the art must change

### NHST is unsuitable as the cornerstone of scientific inquiry in most fields

In summary, NHST provides *the illusion of certainty* through supposedly ‘objective’ binary accept/reject decisions (Cohen, 1994; Ioannidis, 2012) based on practically not very useful *p*-values (Bakan, 1966). However, researchers usually never give any formal assessment of how well their theory (a specific H_1_) fits the facts and, instead of gradual model building (Gigerenzer, 1998) and comparing the plausibility of theories, they can get away with destroying a strawman: they disprove an H_0_ (which happens inevitably sooner or later) with a machinery biased to disproving it without ever going into much detail about the *exact* behavior of variables under *exactly* specified hypotheses (Kranz, 1999; Jaynes, 2003). NHST also does not allow for systematic knowledge accumulation. In addition, both because of its shortcomings and because it is subject to major misunderstandings it facilitates the production of non-replicable false positive reports. Such reports ultimately erode scientific credibility and result in wasting perhaps most of the research funding in some areas (Ioannidis, 2005; Macleod et al., 2014; Kaplan and Irvin, 2015; Nosek et al., 2015).

NHST seems to dominate biomedical research for various reasons. First, it allows for the easy production of a large number of publishable papers (irrespective of their truth value) providing a response to publication pressure. Second, NHST seems deceptively simple: because the burden of inference (Bakan, 1966) has been delegated to the significance test all too often researchers' statistical world view is narrowed to checking an inequality: is *p* ≤ 0.05 (Cohen, 1994)? After passing this test, an observation can become a “scientific fact” contradicting the random nature of statistical inference (Gelman, 2015). Third, in biomedical and social science NHST is often falsely perceived as the *single* objective approach to scientific inference (Gigerenzer et al., 1989) and alternatives are simply not taught and/or understood.

We have now decades of negative experience with NHST which gradually achieved dominance in biomedical and social science since the 1930s (Gigerenzer et al., 1989). Critique of NHST started not much later (Jeffreys, 1939, 1948, 1961) and has been forcefully present since then (Jeffreys, 1939, 1948, 1961; Eysenck, 1960; Nunnally, 1960; Rozeboom, 1960; Clark, 1963; Bakan, 1966; Meehl, 1967; Lykken, 1968) and continues to-date (Wasserstein and Lazar, 2016). The problems are numerous, and as Edwards (1972, p. 179) concluded 44 years ago: “*any method which invites the contemplation of a null hypothesis is open to grave misuse, or even abuse*.” Time has proven this statement and that problems are unlikely to go away. We suggest that that it is *really* time for change now.

### When and how to use NHST

Importantly, we do not want to ban NHST (Hunter, 1997), we realize that it may be reasonable to use it in some well-justified cases. In all cases when NHST is used its use must be justified clearly rather than used as an automatic default and single cornerstone procedure. On the one hand, NHST can be used when very precise quantitative theoretical predictions can be tested, hence, both power and effect size can be estimated well as intended by Neyman and Pearson (1933). On the other hand, when theoretical predictions are not precise, reasonably powered NHST tests may be used as an initial heuristic look at the data as Fisher (1925) intended. However, in these cases (when well-justified theoretical predictions are lacking) if studies are not pre-registered (see below) NHST tests can only be considered preliminary (exploratory) heuristics. Hence, their findings should only be taken seriously if they are replicated, optimally within the same paper (Nosek et al., 2013). These replications must be well powered to keep FRP low. As discussed, NHST can only reject H_0_ and can accept neither a generic or specific H_1_. So, on its own NHST cannot provide evidence “for” something even if findings are replicated.

For example, if initially researches do not know where to expect experimental effects in a particular experimental task, they could run a whole brain, multiple-testing corrected search for statistical significance in a group of participants. Such a search would provide heuristic evidence if they identify some brain areas reacting to manipulations. In order to confirm these effects they would need to carefully study for example the BOLD signal or EEG amplitude changes in areas or over electrodes of interest, make predictions about the behavior of these variables, replicate measurements and minimally confirm the previous NHST results before the findings can be taken seriously. Much better, if researchers can also provide some model for the behavior of their variables, make model predictions and then confirm these with likelihood-based and/or Bayesian methods. Making such predictions would probably require intimate familiarity with a lot of raw data.

### Ways to change

In most biomedical, neuroscience, psychology, and social science fields currently popular analysis methods are based on NHST. It is clear that analysis software and researcher knowledge cannot be changed overnight. Below we summarize some further recommendations which we think can minimize the negative features of NHST even if it continues to be dominant for a while. A very important practical goal would be to change the incentive structure of biomedical and social science to bring it in line with these and similar other recommendations (Wagenmakers et al., 2011; Begley and Ellis, 2012; Nosek et al., 2013; Stodden et al., 2016). Also note that we are not arguing against statistical inference which we consider the “logic of science” (Jaynes, 2003; p. xxii.), quantitative and well justified statistical inference should be at the *core* of the scientific enterprise.

#### If theory is weak, focus on raw data, estimating effect sizes, and their uncertainty

The currently dominant, NHST influenced approach is that instead of understanding raw data researchers often just focus on the all or nothing rejection of a vaguely defined H_0_ and shift their attention to interpreting brain “activations” revealed by potentially highly misleading statistical parameter maps. Based on these maps then strong (qualitative) claims may be made about alternative theories whose support may in fact never be tested. So, current approaches seem to reward exuberant theory building based on small and underpowered studies (Szucs and Ioannidis, 2017) much more than meticulous data collection and understanding and modeling extensive raw data patterns. For analogy, in astronomy theories typically built on thousands of years of sky observation data open to everyone. For example, Kepler could identify the correct laws of planetary motion because he had access to the large volume of observational data accumulated by Tycho Brache who devoted decades of his life to much more precise data collection than previously done. Similarly, the crucial tests of Einstein's theories were precise predictions about data which could be verified or falsified (Smolin, 2006; Chaisson and McMillan, 2017).

Overall, if we just consider competing “theories” without ever deeply considering extensive raw data patterns it is unlikely that major robust scientific breakthroughs will be done whereas many different plausible looking theories can be promoted. Imagine, for example, a situation where astronomers would have only published the outcomes of their NHST tests, some rejecting that the sun is in the middle of the universe while others rejecting that the earth is in the middle of the universe while publishing no actual raw data. Meta-analyses of published effect sizes would have confirmed both positions as both camps would have only published test statistics which passed the statistical significance threshold. Luckily, real astronomers recorded a lot of data and derived testable theories with precise predictions.

In basic biomedical and psychology research we often cannot provide very well worked out hypotheses and even a simple directional hypothesis may seem particularly enlightening. Such rudimentary state of knowledge can be respected. However, in such pre-hypothesis stage substantively blind all or nothing accept/reject decisions may be unhelpful and may maintain our ignorance rather than facilitate organizing new information into proper quantitative scientific models. It is much more meaningful to focus on assessing the magnitude of effects along with estimates of uncertainty, let these be error terms, confidence intervals or Bayesian credible intervals (Edwards, 1972; Luce, 1988; Schmidt, 1996; Jaynes, 2003; Gelman, 2013a,b; see Morey et al., 2016 on the difference between classical confidence intervals and Bayesian credible intervals). These provide more direct information on the actual “empirical” behavior of our variables and/or the precision of interval estimation. Gaining enough experience with interval estimates and assuring their robustness by building replication into design (Nosek et al., 2013) may then allow us to describe the behavior of variables by more and more precise scientific models which may provide more clear predictions (Schmidt, 1996; Jaynes, 2003; Gelman, 2013a,b).

The above problem does not only concern perceived “soft areas” of science where measurement, predictions, control and quantification are thought to be less rigorous than in “hard” areas (Meehl, 1978). In many fields, for example, in cognitive neuroscience, the measurement methods may be “hard” but theoretical predictions and analysis often may be just as “soft” as in any area of “soft” psychology: Using a state of the art fMRI scanner for data collection and novel but extremely complicated and often not well understood analysis paths will not make a badly defined theory well-defined.

The change of emphasis suggested here would require that instead of *p*-values and reporting the outcomes of all/nothing hypothesis tests studies should focus on reporting data in original units of measurement as well as providing derived effect sizes. It is important to publish data summaries (means, standard errors, nowadays extremely rarely plotted empirical data distributions) in original units of measurement as derived measures may be highly biased by some (undocumented) analysis techniques. If we have clear and pre-registered hypotheses then it is relatively straightforward to publish raw data summaries (e.g., mean BOLD signal or ERP amplitude change with standard errors) related to those hypotheses. Clear data presentation usually gets difficult when there are lots of incidental findings. Usually unlimited amount of data summaries can now be published cost free in Supplementary Materials.

#### Pre-registration

In our view one of the most important and virtually cost-free (to researchers) improvement would be to pre-register hypotheses and analysis parameters and approaches (in line with Section 5.2 in Nichols et al., 2016; p11; Gelman and Loken, 2014). Pre-registration can easily be done for example, at the website of the Open Science Foundation (osf.org), also in a manner that it does not immediately become public. Hence, competitors will not be able to scoop good ideas before the study is published. Considering the extreme analysis flexibility offered by high-dimensional neuroscience data (Kriegeskorte et al., 2009; Vul et al., 2009; Carp, 2012) pre-registration seems a necessary pre-condition of robust hypothesis driven neuroscience research. Pre-registration would likely help to cleanse non-replicable “unchallenged fallacies” (Ioannidis, 2012) from the literature. For example, Kaplan and Irvin (2015) found that pre-registering the primary hypotheses of clinical studies decreased the proportion of positive findings from 57% (17 of 30 studies) to 8% (2 out of 25 studies). Hence, another benefit of pre-registration would be to decrease publication volumes. This would require changing the incentive system motivating scientists (Nosek et al., 2013).

It is to note that honesty regarding pre-registration and challenging questionable research practices (Simmons et al., 2011) is the shared responsibility of all co-authors. Some fields in medical research have already over 10 years of experience with pre-registration. This experience shows that pre-registration needs to be thorough to be reliable. For example, many observational studies claimed to have been registered but closer scrutiny shows that registration has actually happened after the study/analysis was done (Boccia et al., 2016). In other cases, clinical trials may be seemingly properly pre-registered before they start recruiting patients, but analyses and outcomes were still manipulated after registration (Ioannidis et al., 2017). Hence, proper safeguards should be put in place to ensure that scientists are accountable for any misconduct regarding breaching pre-registration rules.

#### Publish all analysis scripts with analysis settings

Another cost-free improvement is to publish all analysis scripts with the ability to regenerate all figures and tables (Laine et al., 2007; Peng, 2009, 2011; Diggle and Zeger, 2010; Keiding, 2010; Doshi et al., 2013). This does not require large storage space and can also be done in Supplementary Material. If researchers keep this expectation in mind from the start of a project then implementing it becomes relatively straightforward. Program code will often provide information which is missing from papers. With regard to missing analysis information it is important to be conscious of the fact that seemingly innocuous and irrelevant analysis settings (e.g., slightly changing initial filtering parameters) can have major impact on final statistical outcomes at the end of a complicated processing pipeline. For example, modified initial settings may change the statistically significant/non-significant status of final important test statistics. This can be an issue if multiple settings can be justified and/or if some settings leading to significant outcomes are actually less justified than alternative settings. Publishing scripts will also provide more information on potential statistical errors (Bakker and Wicherts, 2001; Nuijten et al., 2016).

It is to note that in-house analysis scripts may provide substantial competitive advantage to researchers who are able to programme these. Hence, the unconditional release of these scripts may deprive researchers from an important competitive asset. In such cases in house scripts could be documented in brief methods papers which could be cited when the relevant scripts are used so that researchers benefit from citations. Perhaps specialized methods repository journals could be set up for this purpose. For some recent recommendations for improving computational reproducibility practices see Stodden et al. (2016).

#### Publish raw data

In an ideal world researchers should publish all raw data. This is easy with small volumes of behavioral data but it has serious monetary and time investment costs with large neural data volumes (see also Nichols et al., 2017). Some repositories have already been set up and it is important that funders cover these costs and optimally provide infrastructure (see Pernet and Poline, 2015; Nichols et al., 2017). Incentives such as a badge system may help promote availability of more raw data (Nosek et al., 2015). In our opinion it is important to publish unprocessed *raw* data because processed data may already have been distorted/biased in undocumented ways. In general, it is more and more usual to reanalyse data from large repositories, so much further development can be expected in this area (e.g., Eklund et al., 2012).

#### Publish data (summaries) irrespective of statistical significance, promote building good quality datasets including large replication studies

It is important to publish data summaries and/or data sets, including the ones not resulting in statistically significant findings. Without these datasets true effect sizes simply cannot be determined. This will require that these datasets become citeable so that their authors can be rewarded if data is used for secondary analyses. Considering for example the above mentioned case of Tycho Brache it is clear that his data collection exercise was a necessary precondition of crowning the Copernican/Newtonian revolution of astronomy (Chaisson and McMillan, 2017). Hence, we should be able to reward the mere collection of large volumes of good quality data: such activity can prove to be an immense service to the whole profession. Initiatives, like registered multi-lab replication studies should also be prioritized when the validity of important proposals is at stake. Funders are currently often reluctant to fund such studies. However, they should realize that the continuous seeking of new results and theories may just waste most of their resources (Ioannidis et al., 2014; Kaplan and Irvin, 2015).

#### Increase statistical power and publish pre-study power calculations

In real world research it is usually impossible to determine the statistical power of NHST tests exactly. However, if raw data summaries and/or raw data is published irrespective of statistical significance (Section Pre-registration, Publish Raw Data, and Publish Data (Summaries) Irrespective of Statistical Significance, Promote Building Good Quality Datasets Including Large Replication Studies) then we have a much better chance of trying to determine power. Another option is to determine power to detect pre-defined standardized effect sizes. In any case, power in psychology and neuroscience should be much higher than what it is nowadays (Szucs and Ioannidis, 2017). We hypothesize that pre-registration would facilitate increasing power because researchers could less expect to rely on incidentally finding something statistically significant to report from their studies. Hence, they would have more interest in assuring that they are able to respond their primary, registered hypotheses.

#### Better training and better use of more statistical methods: from believers to thinkers

A core problem seems to be that the statistical subject knowledge of many researchers in biomedical and social science has been shown to be poor (Oakes, 1986; Gliner et al., 2002; Castro Sotos et al., 2007, 2009; Wilkerson and Olson, 2010; Hoekstra et al., 2014). NHST perfectly fits with poor understanding because of the perceived simplicity of interpreting its outcome: is *p* ≤ 0.05 (Cohen, 1994)?

We suggest that the weak statistical understanding is probably due to inadequate “statistics lite” education. This approach does not build up appropriate mathematical fundamentals and does not provide scientifically rigorous introduction into statistics. Hence, students' knowledge may remain imprecise, patchy, and prone to serious misunderstandings. What this approach achieves, however, is providing students with false confidence of being able to use inferential tools whereas *they usually only interpret the p-value provided by black box statistical software*. While this educational problem remains unaddressed, poor statistical practices will prevail regardless of what procedures and measures may be favored and/or banned by editorials.

All too often statistical understanding is perceived as something external to the subject matter of substantive research. However, it is important to see that statistical understanding influences most decisions about substantive questions, because it underlies the *thinking* of researchers even if this remains *implicit*. While common sense “statistics” may be able to cope with simple situations, common sense is not enough to decipher scientific puzzles involving dozens, hundreds, or even thousands of interrelated variables. In such cases well justified applications of probability theory are necessary (Jaynes, 2003). Hence, instead of delegating their judgment to “automatized” but ultimately spurious decision mechanisms, researchers should have confidence in their own *informed judgment* when they make an inference. Such confidence requires deep study.

Understanding probability is difficult. Common sense is notoriously weak in understanding phenomena based on probabilities (Gigerenzer et al., 2005). We cannot assume that without proper training biomedical and social science graduates would get miraculously enlightened about probability. Some of the best symbolic thinking minds of humanity devoted hundreds of years to the proper understanding of probability and statisticians still do not agree on how best to draw statistical inference (Stigler, 1986; Gigerenzer et al., 1989), e.g., the recent American Statistical Association statement on *p*-values (Wasserstein and Lazar, 2016) was accompanied by 21 editorials from the statisticians and methodologists who participated in crafting it and who disagreed in different aspects among themselves.

There is no reason to assume that understanding twenty first and twenty second century science will require less mathematical and statistical understanding than before. Such as there is no royal road to mathematics, there is no royal road to statistics which is heavily based on mathematics. If statistical understanding does not improve it will not matter whether editorials enforce bootstrapping, likelihood estimation or Bayesian approaches, they will all remain opaque to the untrained mind and open to abuse such as the NHST of the twentieth century.

One approach would be to phase out the ‘statistics lite’ education approach for all research stream students and teach statistics rigorously. A typical research stream undergraduate training could include, for example, 3–4 semesters of calculus, one semester of introductory statistics, three more semesters of calculus based statistics, and then finally two semesters of more specialized statistics. An alternative and/or complementary approach would be to enhance the training of professional applied statisticians and to ensure that all research involves knowledgeable statisticians or equivalent methodologists. At a minimum, all scientists should be well trained in understanding evidence and statistics and being in a position to recognize that they may need help from a methodologist expert (Marusic and Marusic, 2003; Moharari et al., 2009; Vujaklija et al., 2010).

#### Teach alternative approaches seriously

It is important that researchers are conscious that NHST only represents a small segment of available statistical techniques. Besides NHST, Bayesian and likelihood based approaches should also be taught, with explanation of the strengths and weaknesses of each inferential method. Hypotheses could be tested by either likelihood ratio testing, and/or Bayesian methods which usually view probability as characterizing the state of our beliefs about the world (Pearl, 1988; Jaynes, 2003; MacKay, 2003; Sivia and Skilling, 2006; Gelman et al., 2014; for neuroscience data see e.g., Lorenz et al., 2017). The above alternative approaches typically require model specifications about alternative hypotheses, they can give probability statements about H_0_ and alternative hypotheses, they allow for clear model comparison, are insensitive to data collection procedures and do not suffer from problems with large samples. In addition, Bayesian methods can also factor in pre-study (prior) information into model evaluations which may be important for integrating current and previous research findings. Hence, the above alternative approaches seem more suitable for the purpose of scientific inquiry than NHST and ample literature is available on both. The problem is that usually none of these alternative approaches are taught properly in statistics courses for students in psychology, neuroscience, biomedical science and social science. For example, across 1,000 abstracts randomly selected from the biomedical literature of 1990–2015, none reported results in a Bayesian framework (Chavalarias et al., 1990-2015).

It is important to note that Bayesian methods are often accused of subjectivity because they can take prior information into account. However, Bayesian methods are able to consider prior expectations formally and explicitly in their models provided that necessary information (e.g., raw data and/or extensive reporting of data parameters) from previous studies is available. In contrast, as we have discussed NHST can be latently biased by subjectivity at many points without ever revealing any of the biases. In contrast, different reasonable Bayesian priors can be implemented and their impact on outcomes can be debated explicitly and ultimately, the goodness of model predictions can be tested. Hence, we do not see the use of Bayesian priors as a drawback. Rather, explicit priors can represent a strength as they allow for formal knowledge integration from previous studies.

### There is no automatic inference: new-old dangers ahead?

Perhaps the most worrisome false belief about statistics is the belief in automatic statistical inference (Bakan, 1966; Gigerenzer and Marewski, 1998), the illusion that plugging in some numbers into some black box algorithm will give a number (perhaps the *p*-value or some other metric) that conclusively proves or disproves hypotheses (Bakan, 1966). There is no reason to assume that any kind of “new statistics” (Cumming, 2014) will not suffer the fate of NHST if statistical understanding is inadequate. For example, it has been shown that confidence intervals are misinterpreted just as badly as *p*-values by undergraduates, graduates, and researchers alike and self-declared statistical experience even slightly positively correlates with the number of errors (Hoekstra et al., 2014). Or, many times black box machine learning algorithms may be run uncritically and/or on relatively small data volumes. However, the more complex is a dataset the more chance such substantively blind search algorithms have to find some relationships where nothing worthy of mention exist. So, uncritical applications are likely to further boost the proportion of false positive findings irrespective of the sophistication of the algorithms (Skokic et al., 2016). Similarly, the proper use of Bayesian methods may require use of advanced simulation methods and a clear understanding and justification of probability distribution models. In contrast to this, it is frequent to see a kind of “automatic” determination of Bayes factors or posterior estimates, again, provided by black box statistical packages which again, promise to take the load of thinking off the shoulders of researchers.

## Author note

A previous version of this manuscript was available as a pre-print (http://biorxiv.org/content/early/2016/12/20/095570). The copyright holder for this preprint is the author/funder. It is made available under a CC-BY-NC-ND 4.0 International license.

## Author contributions

DS wrote the first draft of the manuscript. DS and JI revised successive drafts.

### Conflict of interest statement

The authors declare that the research was conducted in the absence of any commercial or financial relationships that could be construed as a potential conflict of interest.
