# Supplementary material for: When Null Hypothesis Significance Testing Is Unsuitable for Research: A Reassessment
Source: Front Hum Neurosci. 2017 Aug 3;11:390. doi: 10.3389/fnhum.2017.00390 (PMC5540883; doi:10.3389/fnhum.2017.00390)
Supplement: Supplementary file 1 [file Presentation1.PDF]

Appendix 1.

Definitions of statistical terms.

Null Hypothesis (H_0): A hypothesis set up with the intention of falsifying (nullifying) it. In most NHST studies H_0 typically expects that there is no experimental effect (zero sized effect).

Alternative Hypothesis (H_1): A generic alternative hypothesis expects *some* kind of experimental effect, typically non-zero difference between treatment levels and/or groups. In principle, the general H_1 is the complement of H_0. However, in practice such generic H_1 is so hard to define that it may be useless in practical terms (Hubbard and Bayarri, 2003; Jaynes, 2003). So, while H_1 refers to a *general* alternative hypothesis (the complement of H_0) most practicing researchers have a *specific* H_1 in mind.

P value: The p value (p) is one possible continuous measure of the discrepancy between a particular model, called null hypothesis or H_0, and the data (Gelman 2013). More specifically, p is the probability of observing the experimental data or more extreme data under the assumption that H_0 is correct. In yet another way, we can link the p value to the test statistic: The p value is the probability that a test statistic, computed on the assumption that H_0 is true, would take a value as extreme or more extreme than the one observed (Murdoch et al. 2008). The p value is a random variable that depends on sample size, effect size and random sampling variation in the data and is also affected by potential biases. Hence, two studies measuring identical underlying effect sizes with the same sample size can find different p values.

Type I error: False positive error. The error of mistakenly rejecting H_0 and therefore reporting a statistically significant finding when H_0 is really true. This Type I error rate can be controlled by the α level set by the experimenter.

Alpha (α) level: α is the *long run* probability of committing a Type I error. α is the critical p value under which the test result is declared to be 'statistically significant' ($p \leq \alpha$) . Typically $\alpha = 0.05$ which means that *on the long run* $100\alpha\% = 5\%$ of findings will be statistically significant *provided* that H_0 is really true. The Type I error rate is fix on the long run irrespective of the sample size and the p value found in a study. Note that setting α to any particular level is completely arbitrary and it does not have any mathematical justification.

Complementary α level (1-α); confidence level: The long run probability of retaining H_0 if H_0 is really true. E.g. if $\alpha = 0.05$ then the confidence level is $1 - \alpha = 0.95$.

Type II error: False negative error. The error of mistakenly not rejecting H_0 and therefore reporting a statistically non-significant finding when H_1 is really true. Provided that we know exactly the expected effect size Type II error can be controlled by setting the sample size.

β: The long run probability of committing a Type II error assuming that a *particular* H_1 with a particular effect size and probability distribution is true (ie. H_0 can be rejected). β is the complement of Power, that is, $\beta = 1$ - Power.

Power: The power of a study is the *long-run* probability of detecting true positive findings if they really exist given that H_1 has a particular probability distribution and a particular effect size. Power is a function of the sample size and effect size. Typically, Power ≥ 0.8 is considered optimal. This means that if there is an experimental effect equal to or larger than the expected effect size (H_1 is true) then using a given sample size researchers would have 80% chance to identify this true effect in a long run of experiments.

Odds: The ratio of probabilities. For example, if the probability of H_0 is $pr(H_0) = 9/10$ and $pr(H_1) = 1/10$ then the H_0:H_1 odds are: $9/10 : 1/10 = 9:1 = 9$. Conversely, if the odds of H_0:H_1 data are 9, ie. 9:1 than $pr(H_0)=9/(9+1) = 9/10$; and $p(H_1) = 1/(9+1) = 1/10$.

Prior probability of a hypothesis: The pre-study probability of the truth of a hypothesis *without* taking into account any information from the data of the actual study. Note that the term 'prior' only refers to the fact that $p(H_0)$ is independent from study results and does not necessarily refer to temporal precedence (Jaynes, 2003).

FRP: False report probability: The long run probability of H_0 being true given a significant result with particular α and β levels, with particular Odds of true positive and negative results and with particular experimenter Bias in a long series of experiments.

TRP: True report Probability: The long run probability of H_1 being true given a significant result with particular α and β levels, with particular Odds of true positive and negative results and with particular experimenter Bias in a long series of experiments.

References are listed in the main text.

Appendix 2.
Major confusions about the p value.

1. Many practicing researchers and even some statisticians confuse the roles of the p value and α (Hubbard and Bayarri 2003). These researchers set a significance level of $\alpha = 0.05$ before they run an experiment but once they compute the p value they falsely assume that the p value will now represent the actual data-dependent Type I error probability somehow replacing the Neyman-Pearson α level while also interpreting it as the strength of evidence against H_0 as used by Fisher (Goodman, 1993; 1999; Nickerson 2000). However, α is always fixed independently of what p value we find in an experiment whereas p values can be considered random variables, varying widely from experiment to experiment (Murdoch et al. 2008; Hung et al. 1997; Simonsohn et al. 2014a,b; Sterling 1959). Currently, the expression 'significance level' is used interchangeably for both the p value and α reflecting the confusion about them (Hubbard and Bayarri 2003).

2. Many practicing researchers falsely assume that if $p = 0.01$ then the probability of a false positive finding given the data ($pr(H_0|data)$ is 0.01. Conversely, they also assume that if $p = 0.01$ then the probability of a truly positive finding given the data ($pr(H_1|data)$ is $1 - p = 0.99$. Yet, others confuse the p value with the 'updated' H_0:H_1 odds after a study was run, and/or with replication success (Bakan, 1966; Meehl, 1967; Pollard and Richardson, 1987; Cohen 1994; Hunter, 1997; Goodman, 1999; Oakes, 1986; Gliner et al. 2002; Wilkerson and Olson, 2010; Hoekstra et al. 2014; Castro-Sotos 2007; 2009). These *false* assumptions are not only *thoroughly wrong*, they also deeply *underestimate* the probability of false positive findings and highly *overestimate* the probability of truly positive findings and replication success. The network of confusions outlined here constitute what Goodman (1999) termed the *'p value fallacy'* (see Goodman, 1999; Goodman 2008; Nickerson 2000 and Wagenmakers, 2007 for excellent reviews).

References are listed in the main text.

Appendix 3.

False report probability and True report probability

For the convenience of the reader this Appendix restates part of the Supplementary Material from Szucs and Ioannidis (2017) PLoS Biology; 15(3): e2000797; 2 March 2017.

If we use nil-null Hypothesis Significance Testing (NHST) then the long run False Report Probability (FRP) is the long run probability that the null hypothesis (H_0) is true when we get a statistically significant finding. The long run True Report Probability (TRP) is the long run probability that the alternative hypothesis (H_1) is true when we get a statistically significant finding. Computationally, FRP is the number of statistically significant false positive findings divided by the total number of statistically significant findings. TRP is the number of statistically significant truly positive findings divided by the total number of statistically significant findings.

Calculations are set out below. **Figure 3 and Supplementary Figure 1** provide graphical illustrations.

FRP and TRP can be computed by Bayes' theorem. If we take 'sig' to stand for 'statistically significant test outcome' then the total probability of finding a statistically significant result is:

$$\mathrm{pr(sig)} = \mathrm{pr(sig|H_0)} * \mathrm{pr(H_0)} + \mathrm{pr(sig|H_1)} * \mathrm{pr(H_1)} \;\; ; (Eq.1)$$

Hence, FRP and TRP can be written as:

$$\mathrm{FRP} = \frac{\mathrm{pr(sig|H_0)} * \mathrm{pr(H_0)}}{\mathrm{pr(sig)}} \;\; ; (Eq.2)$$

$$\mathrm{TRP} = \frac{\mathrm{pr(sig|H_1)} * \mathrm{pr(H_1)}}{\mathrm{pr(sig)}} \;\; ; (Eq.3)$$

Considering a long run of NHST studies, the long run probability of having a significant test outcome when H_0 is true is α and the long run probability of having a significant test outcome when H_1 is true is Power = 1 - β. That is, $\alpha = \mathrm{pr(sig|H_0)}$ and Power = $\mathrm{p(sig|H_1)}$. Hence, **Eq.2.** and **Eq.3.** can be re-written as:

$$\mathrm{FRP} = \frac{\alpha * \mathrm{pr(H_0)}}{\mathrm{pr(sig)}} \;\; ; (Eq.4)$$

$$\mathrm{TRP} = \frac{\mathrm{Power} * \mathrm{pr(H_1)}}{\mathrm{pr(sig)}} \;\; ; (Eq.5)$$

Eq.2. and **Eq.3.** can also be expressed in terms of odds ratios of true H_0 and true H_1 situations. For example, we can denote the odds of true H_0 situations as 'O' and write:

$$\mathrm{O} = \frac{pr(H_0)}{pr(H_1)} \; ; (Eq.6)$$

We can express $\mathrm{pr(H_0)}$ using the above as:

$$\mathrm{pr}(H_0) = O * \mathrm{pr}(\mathrm{H}_1) \;\; ;(Eq.7)$$

then **Eq.2.** Can be rewritten as:

$$\mathrm{FRP} = \frac{\mathrm{pr}(\mathrm{sig}|\mathrm{H}_0) * \mathrm{O} * \mathrm{pr}(\mathrm{H}_1)}{\mathrm{pr}(\mathrm{sig}|\mathrm{H}_0) * \mathrm{O} * \mathrm{p}(\mathrm{H}_1) + \mathrm{pr}(\mathrm{sig}|\mathrm{H}_1) * \mathrm{pr}(\mathrm{H}_1)} \;\; ;(Eq.8)$$

This can be simplified by $\mathrm{pr}(\mathrm{H}_1)$:

$$\mathrm{FRP} = \frac{\mathrm{pr}(\mathrm{sig}|\mathrm{H}_0) * Odds_{H0}}{\mathrm{pr}(\mathrm{sig}|\mathrm{H}_0) * Odds_{H0} + \mathrm{pr}(\mathrm{sig}|\mathrm{H}_1)} \;\; ;(Eq.9)$$

Using α and Power = 1 - β we can write:

$$\mathrm{FRP} = \frac{\mathrm{O}\alpha}{\mathrm{O}\alpha + \mathrm{Power}} \;\; ;(Eq.10)$$

$$\mathrm{TRP} = \frac{\mathrm{Power}}{\mathrm{O}\alpha + \mathrm{Power}} \;\; ;(Eq.11)$$

FRP + TRP = 1; e.g.:

$$\frac{\mathrm{O}\alpha}{\mathrm{O}\alpha + \mathrm{Power}} + \frac{Power}{\mathrm{O}\alpha + Power} = \frac{\mathrm{O}\alpha + Power}{\mathrm{O}\alpha + Power} = 1 \;\; (Eq.12)$$

Consequently:

$$\mathrm{FRP} = 1 - \mathrm{TRP} \;\; (Eq.13)$$

and

$$\mathrm{TRP} = 1 - \mathrm{FRP} \;\; (Eq.14)$$

Equivalently to the above, we can also express the odds of true H_1 situations as the ratio of $pr(H_1)$ and $pr(H_0)$ and denote it with 'R' as in Ioannidis (2005):

$$R = \frac{pr(H_1)}{pr(H_0)} \;\; ;(Eq.15)$$

$$\mathrm{pr}(H_1) = R * \mathrm{pr}(H_0) \;\; ;(Eq.16)$$

Substituting **Eq.16.** into **Eq.3.**:

$$\mathrm{TRP} = \frac{\mathrm{pr}(\mathrm{sig}|\mathrm{H}_1) * R * \mathrm{pr}(\mathrm{H}_0)}{\mathrm{pr}(\mathrm{sig}|\mathrm{H}_0) * \mathrm{p}(\mathrm{H}_0) + \mathrm{pr}(\mathrm{sig}|\mathrm{H}_1) * \mathrm{R} * \mathrm{pr}(\mathrm{H}_0)} \;\; ;(Eq.17)$$

Simplifying:

$$\mathrm{TRP} = \frac{\mathrm{pr}(\mathrm{sig}|\mathrm{H}_1) * R}{\mathrm{pr}(\mathrm{sig}|\mathrm{H}_0) + \mathrm{R} * Odds_{H1}} \;\; ;(Eq.18)$$

Using α and Power = 1 - β we can write:

$$\mathrm{TRP} = \frac{\mathrm{R} * \mathrm{Power}}{\alpha + \mathrm{R} * \mathrm{Power}} \;\; ;(Eq.19)$$

$$\mathrm{FRP} = \frac{\alpha}{\alpha + \mathrm{R} * \mathrm{Power}} \; ; (Eq. 20)$$

Eq.19. is equivalent to the one used by Ioannidis (2005) with a slightly different notation.

He defined PPV = TRP; Power = 1-β and equivalently to Eq. 19 he wrote:

$$\mathrm{PPV} = \frac{(1-\beta) * \mathrm{R}}{\alpha + (1-\beta) * R} = \frac{(1-\beta) * \mathrm{R}}{\alpha + \mathrm{R} - \beta R} \; ; (Eq. 21)$$

Ioannidis (2005) also defined Bias, signified by '*u*'. On the one hand, bias results in categorizing fraction u (u*(1 - α)) of otherwise true negative results (in case there is no bias) as positive results. On the other hand, bias results in categorizing fraction *u* (uβ = u*(1 - Power)) of otherwise (in case there is no bias) missed true positive results as positive results. That is, bias alters **Eq.19.** as:

$$\mathrm{TRP} = \frac{\mathrm{Power} * R + u * (1 - \mathrm{Power}) * \mathrm{R}}{\alpha + \mathrm{u} * (1 - \alpha) + \mathrm{Power} * \mathrm{R} + u * (1 - \mathrm{Power}) * \mathrm{R}} \; ; (Eq. 22)$$

Using the notation of Ioannidis (2005) this can be rewritten as:

$$\mathrm{PPV} = \frac{(1-\beta) * \mathrm{R} + \mathrm{u}\beta\mathrm{R}}{\alpha + \mathrm{u}(1-\alpha) + (1-\beta) * R + u\beta\mathrm{R}}$$
$$= \frac{(1-\beta) * \mathrm{R} + \mathrm{u}\beta\mathrm{R}}{\alpha + \mathrm{u} - \alpha\mathrm{u} + \mathrm{R} - \beta R + u\beta\mathrm{R}} \; ; (Eq. 23)$$

Also, notice the relation between O and R:

$$O = \frac{p(H_0)}{p(H_1)} = \frac{1}{R} \; ; \; (Eq. 24)$$

Hence,

$$\mathrm{FRP} = \frac{\alpha * \frac{1}{R}}{\alpha * \frac{1}{R} + \mathrm{Power}} = \frac{\frac{\alpha}{R}}{\frac{\alpha + \mathrm{Power} * R}{R}} = \frac{\alpha}{\alpha + \mathrm{Power} * R} ; (Eq. 25)$$

Similarly:

$$R = \frac{p(H_1)}{p(H_0)} = \frac{1}{O} \; ; \; (Eq. 26)$$

Hence,

$$\mathrm{TRP} = \frac{\mathrm{Power} * \frac{1}{O}}{\alpha + \mathrm{Power} * \frac{1}{\mathrm{O}}} = \frac{\frac{Power}{\mathrm{O}}}{\frac{\alpha * \mathrm{O} + Power}{O}} = \frac{Power}{\alpha * \mathrm{O} + Power} \; ; (Eq. 27)$$

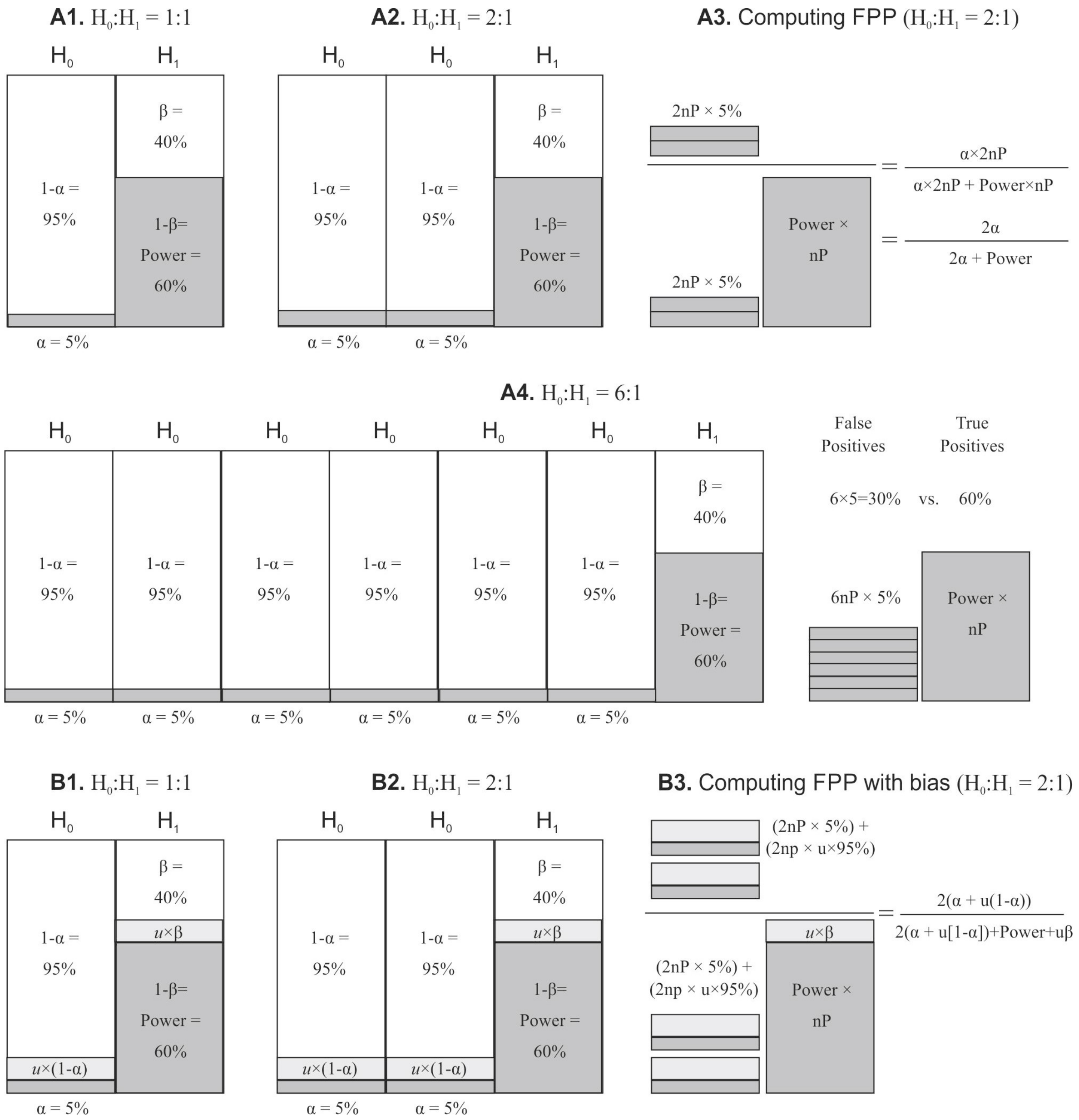

Supplementary Figure 1. ***Conditional probabilities, odds and bias.*** *nP* denotes the total number of studies testing truely positive effects (true H_1). **(A1)** The relations of α, β and power with 1:1 H_0:H_1 odds with α=0.05 and β=0.4 (α and β are kept at these levels in all examples). **(A2)** The relations of α, β and power with 2:1 H_0:H_1 odds. Note that the total number of false positive significant results is now 2nP×α while the total number of truly positive results is Power×nP. **(A3)** Graphical illustration of computing FRP with 2:1 H_0:H_1 odds. FRP is the ratio of false positives divided by the total amount of results coming up significant (false positives + true positives). In the example FRP = 2nP×α / (2nP×α + Power×nP) = 2×0.05 / (2×0.05 + 0.6) = 0.1 / 0.7 = 1/7 = 14.28% rather than the often assumed 1/20, or 5%. **(A4)** Graphical illustration of computing FRP with R=1/6; ie. with 6:1 H_0:H_1 odds. In the example FRP = 6nP×α / (6nP×α + Power×nP) = 6×0.05 / (6×0.05 + 0.6) = 0.3 / 0.9 = 1/3 = 33.33% of all significant results will be false positives. **(B1)** Graphical illustration of bias with 1:1 H_0:H_1 odds (Ioannidis, 2005). Bias increases the proportion of false positives (adds to Type I error) and decreases the proportion of false negatives (adds to power). With realistic H_0:H_1 odds bias will increase the proportion of false positive significant results much faster than it will increase

power (**B2** and **B3)**. Shaded boxes are slightly off scale to enhance the readability of the text.

Appendix 4.

The problem of neglecting H_0:H_1 odds.

NHST logic is based on the so-called *modus tollens*[1] (denying the consequent) argumentation (see **Example A1 in Supplementary Table 1**): It sets up a H_0 model and assumes that if the data fits this model than the test statistic associated with the data should not take more extreme values than a certain threshold (Meehl, 1967; Pollard and Richardson, 1987). If the test statistic contradicts this expectation than NHST assumes that H_0 can be rejected and consequently its complement, H_1 can be accepted. While this logic may be able to minimize Type I error in well powered quality control tests (**2.2**) it is inadequate if we use it to decide about the truth of H_1 in a single experiment (Falk and Greenbaum, 1995). For example, the three non-probabilistic arguments following the above structure in **Supplementary Table 1/A1-3** are correct. However, as soon as we introduce probabilities and modify the arguments accordingly, they become incorrect **(Supplementary Table 1/B1-2).**

For example, let's evaluate **Example B2** from **Supplementary Table 1.** In understanding the problem we can fully follow the long run probability view of NHST. H_0 states that you do not have the mortal disease while H_1 states that you have the mortal disease. Because the data is rare under H_0 NHST logic rejects H_0 and concludes that you have the mortal disease. However, because the statements are probabilistic in some cases where you do not have the mortal disease (H_0 is true) your test result will be ≥ 3 (Type I error). Conversely, in other cases when you do have the mortal disease your test result will be < 3 (Type II error). Type I and Type II errors mean that our conclusions always have some space for error and the only way to see how much error we have on the long run to is calculate the long run FRP and TRP in exactly the same way as in **Section 3** of the main text using appropriate α and power $(1-\beta)$ levels and H_0:H_1 odds. For example, our long run experience may tell us that 100 out of 101 people do not get the disease but 5 of this 100 will test false positive whereas 1 out of 101 has the disease and 99% of them will test positive. In this case the odds of not having the disease are O = 100:1. The false positive identification rate is $\alpha = 0.05$ and the true positive identification rate is power = 0.99. Hence:

---

[1] It is important to notice the asymmetry between the 'denying the consequent' argument (which is valid in a non-probabilistic world) and the 'affirming the consequent' argument which is invalid. This asymmetry leads to the existential vacuum of non-interpretable null results in NHST: while it is possible to reject H_0 (and consequently accept the *complement* of H_0, the general H_1) it is not possible to accept H_0. However, many practicing researchers commit the mistake (Meehl, 1967) and they assume that if their results are non-significant (ie. H_0 cannot be rejected) it follows that the difference between two conditions must be truly zero (H_0 can be accepted). The invalid confirmatory structure is: If A then B. B. Hence, A. (If H_0 than $t<3$. $t<3$. Hence, H_0.) This is invalid because B can be caused by something other than A, the argument does not say that B is exclusively caused by A. For example, even if H_1 is true but we do not have high enough power than our test statistic will not reach high enough values (t remains $t < 3$) so we cannot reject H_0. Taking this example to the extreme, even if H_1were really true, if we kept power deliberately low we could not reject H_0 most of the time but this of course would not mean that we could argue that H_0 was true. The only case when we could definitively accept H_0 is if we had perfect power to detect *any* arbitrarily small deviation from H_0 (power = 1 for each effect size). In that case the test statistic would be significant in every case when the data were not *exactly* zero down to *any* arbitrary precision. This would be equivalent to having this valid conclusion: *If and only if* A then B. B. Hence, A. (It is *sufficient* and *necessary* to have A so that we can have B.).

$$FRP = \frac{False\ positives}{All\ positive\ results} = \frac{O\alpha}{O\alpha\ +\ Power} = \frac{100 \times 0.05}{100 \times 0.05\ +\ 0.6} = .8347.$$

$$TPP\ = 1 - FPP = 0.1653$$

That is, we only have 16.5% probability of having the disease and more than 80% of not having it after such a test in light of long run experience even if we have power = 0.99.

**A. Correct non-probabilistic conclusions**		
**Example A1**	**Example A2**	**Example A3**
Model:<br>If A then B.	*Model:*<br>If Joe is American<br>he *must be* a citizen of the USA.	*Model:*<br>If H_0 is true then<br>it *must be* that $p > \alpha$
Data: Not B. (contradicting the consequence of A)	*Data:*<br>Joe is not the citizen of the USA.	*Data:*<br>$p \leq \alpha$
Hence, not A.	Hence, Joe is not American.	Hence, H_0 is not true.

**B. Incorrect probabilistic conclusions**	
**Example B1**	**Example B2**
Model:<br>If H_0 is true than *most probably* ($p > \alpha$)	*Model:*<br>If you do not have the mortal disease (H_0) than your test score is *most probably* $t < 3$ (on an arbitrary scale).
Data:<br>($p \leq \alpha$)	*Data:*<br>Your test score is $t \geq 3$.
Hence, H_0 is *most probably* not true	Hence, you most probably have the mortal disease (H_0 is rejected; H_1 is accepted).

Supplementary Table 1. Examples for the modus tollens argument. See explanation in text.

Appendix 5.

The problem of neglecting predictions under the alternative hypothesis (H_1).

The example in **Table 2** of the main text follows NHST logic: Our model says that if H_0 is true it is a *very rare* event that Harold is a member of congress. This rare event then happens which is equivalent to finding a small p value. Hence, we conclude that H_0 can be rejected and H_1 is accepted. However, if we carefully explicate all important probabilities it is easy to see that we are being mislead by invalid NHST logic. First, because we have absolutely no idea about Harold's nationality we can set pre-data probabilities of both H_1 and H_0 to 1/2, which means that H_0:H_1 odds are 1:1. Hence, pre-data information is completely uninformative.

$$pr(H_0) = pr(\text{Harold is American}) = \tfrac{1}{2}$$
$$pr(H_1) = pr(\text{Harold is not American}) = \tfrac{1}{2}$$

Then we can explicate the important conditional probabilities of the data (Harold *is* a member of congress) given the possible hypotheses. We can assign arbitrary but plausible probabilities:

$$pr(data|H_0) = pr(\text{Harold is member of congress} \mid \text{American}) = 10^{-7}$$
$$pr(data|H_1) = pr(\text{Harold is member of congress} \mid \text{not American}) = 0$$

Now we can see the problem[2]. That is, while the data is indeed rare under H_0, its probability is actually zero under H_1. So, even if $p \approx 0.0000001$ (because p = pr[data or more extreme data|H_0] rather than pr[data|H_0]), it does not make sense to reject H_0 and accept H_1 because this data just cannot happen if H_1 is true. If we only have these two hypotheses to choose from then it only makes sense to accept H_0 because the data is still possible under H_0 (Jaynes, 2003). We can also explicitly evaluate the probability of H_0 being true with the help of Bayes' theorem in a way which is equivalent to the equations used to evaluate FRP and TRP:

$$pr(H_0|data) = \frac{pr(Harold\ is\ American\ AND\ a\ member\ of\ congress)}{pr(Harold\ is\ a\ member\ of\ congress)}$$
$$= \frac{pr(H_0) \times pr(data|H_0)}{pr(H_0) \times pr(data|H_0) + pr(H_1) \times pr(data|H_1)} = \frac{\frac{1}{2} \times 10^{-7}}{\frac{1}{2} \times 10^{-7} + \frac{1}{2} \times 0}$$
$$= \frac{\frac{1}{2} \times 10^{-7}}{\frac{1}{2} \times 10^{-7} + 0} = 1$$

Now we see that after assigning probabilities and evaluating them properly we have a numerical response which is completely in line with our intuition. Instead of being able to reject H_0, the post-data probability[3] of H_0 is actually 1.
References are listed in the main text.

---

[2] Note the important advice which NHST neglects: 'Always write down the probability of everything' (Steve Gull; cf. MacKay, 2003; p61).

[3] Note that it is perfectly possible to compute pr(H_0|data) thereby avoiding the existential vacuum of dreaded 'null results' by both Bayesian inference and by maximum likelihood estimation methods.
